# The MEK1/2 Pathway as a Therapeutic Target in High-Grade Serous Ovarian Carcinoma

**DOI:** 10.3390/cancers13061369

**Published:** 2021-03-18

**Authors:** Mikhail S. Chesnokov, Imran Khan, Yeonjung Park, Jessica Ezell, Geeta Mehta, Abdelrahman Yousif, Linda J. Hong, Ronald J. Buckanovich, Akimasa Takahashi, Ilana Chefetz

**Affiliations:** 1 The Hormel Institute, University of Minnesota, Austin, MN 55912, USA; mchesnok@umn.edu (M.S.C.); khan0672@umn.edu (I.K.); ayousif@umn.edu (A.Y.); atakahas@umn.edu (A.T.); 2 Division of Hematology Oncology, Department of Internal Medicine, University of Michigan, Ann Arbor, MI 48109, USA; yeon.park@osumc.edu (Y.P.); jessica.ezell@lmunet.edu (J.E.); buckanovichrj@mwri.magee.edu (R.J.B.); 3 Department of Biomedical Engineering, University of Michigan, Ann Arbor, MI 48109, USA; mehtagee@umich.edu; 4 Division of Gynecologic Oncology, Department of Gynecology and Obstetrics, Loma Linda University School of Medicine, Loma Linda, CA 92350, USA; Lihong@llu.edu; 5 Division of Hematology Oncology, Department of Internal Medicine, University of Pittsburgh, Pittsburgh, PA 15213, USA; 6 Department of Obstetrics and Gynecology, Shiga University of Medical Science, Otsu, Shiga 5202152, Japan; 7 Masonic Cancer Center, Minneapolis, MN 55455, USA; 8 Stem Cell Institute, Minneapolis, MN 55455, USA

**Keywords:** high-grade serous ovarian carcinoma, cancer stem-like cells, MEK1/2, trametinib, proliferation

## Abstract

**Simple Summary:**

High-grade serous ovarian carcinoma (HGSOC) has poor prognosis for patients due to its high rate of recurrence and acquired resistance to therapy. MEK1/2-ERK1/2 signaling pathway that controls cell proliferation and survival is active in the majority of HGSOC cases, but its functional impact is unclear. We suggest that inhibition of MEK1/2 with specific inhibitor trametinib may exert anti-cancer effects upon HGSOC cells. Here we demonstrate that trametinib treatment of HGSOC cells indeed prominently inhibits cell proliferation and tumor growth, and that cisplatin-resistant cells displaying high MEK1/2 activity are particularly sensitive to trametinib. However, we also discovered that trametinib treatment of HGSOC cells has no cytotoxic effects and promotes cancer stem-like characteristics. We therefore suggest to use MEK1/2 inhibitors with other treatment strategies targeting cancer stem-like cells, like aldehyde dehydrogenase 1 inhibition that might show together strong synergy.

**Abstract:**

High-grade serous ovarian carcinoma (HGSOC) is the deadliest of gynecological cancers due to its high recurrence rate and acquired chemoresistance. RAS/MEK/ERK pathway activation is linked to cell proliferation and therapeutic resistance, but the role of MEK1/2-ERK1/2 pathway in HGSOC is poorly investigated. We evaluated MEK1/2 pathway activity in clinical HGSOC samples and ovarian cancer cell lines using immunohistochemistry, immunoblotting, and RT-qPCR. HGSOC cell lines were used to assess immediate and lasting effects of MEK1/2 inhibition with trametinib in vitro. Trametinib effect on tumor growth in vivo was investigated using mouse xenografts. MEK1/2 pathway is hyperactivated in HGSOC and is further stimulated by cisplatin treatment. Trametinib treatment causes cell cycle arrest in G1/0-phase and reduces tumor growth rate in vivo but does not induce cell death or reduce fraction of CD133+ stem-like cells, while increasing expression of stemness-associated genes instead. Transient trametinib treatment causes long-term increase in a subpopulation of cells with high aldehyde dehydrogenase (ALDH)1 activity that can survive and grow in non-adherent conditions. We conclude that MEK1/2 inhibition may be a promising approach to suppress ovarian cancer growth as a maintenance therapy. Promotion of stem-like properties upon MEK1/2 inhibition suggests a possible mechanism of resistance, so a combination with CSC-targeting drugs should be considered.

## 1. Introduction

In the US, ovarian cancer ranks 5th in cancer-related deaths in women, displaying the 5th highest mortality rate [1,2]. About 90% of ovarian cancer cases are epithelial in origin [3] with high grade serous ovarian cancer (HGSOC) being the most common and deadly subtype [2,4,5] (additional information available from The Surveillance, Epidemiology, and End Results (SEER) Program of the National Cancer Institute (NCI), https://seer.cancer.gov/statfacts/html/ovary.html (accessed on 16 December 2020). While most HGSOC tumors initially respond well to platinum-based therapy, about 75% of patients experience disease relapse [4,6,7,8,9] due to acquired resistance to chemotherapeutic agents [4,5,10,11]. Multiple mechanisms of chemoresistance include activation of DNA repair systems, increased drug efflux due to ABCB1 membrane transporter overexpression, changes in drug-specific metabolism, and apoptosis inhibition [10,12,13].

Tumor heterogeneity likely contributes to chemotherapy resistance. Ovarian tumors are heterogeneous on both genomic and cellular levels [14,15]. At the cellular level, analysis of cisplatin-sensitive vs. cisplatin-resistant tumors revealed that resistant cells descend from pre-existing minor subpopulations in the primary tumor [16], which most likely represent cancer stem-like cells (CSCs) [17,18,19,20,21,22,23]. CSCs possess high resistance to cytotoxic and genotoxic effects, are capable of self-renewal, and asymmetric division that generates a progeny of fast-proliferating bulk tumor cells [24]. CSCs possess a high tumorigenic potential and can re-initiate tumor development after chemotherapeutic treatment [18,25,26]. Survival and proliferation of CSCs in various tumors has been shown to be highly dependent on activity of mitogen-activated protein kinase (MAPK) signaling [27,28,29,30].

**Figure 1:**
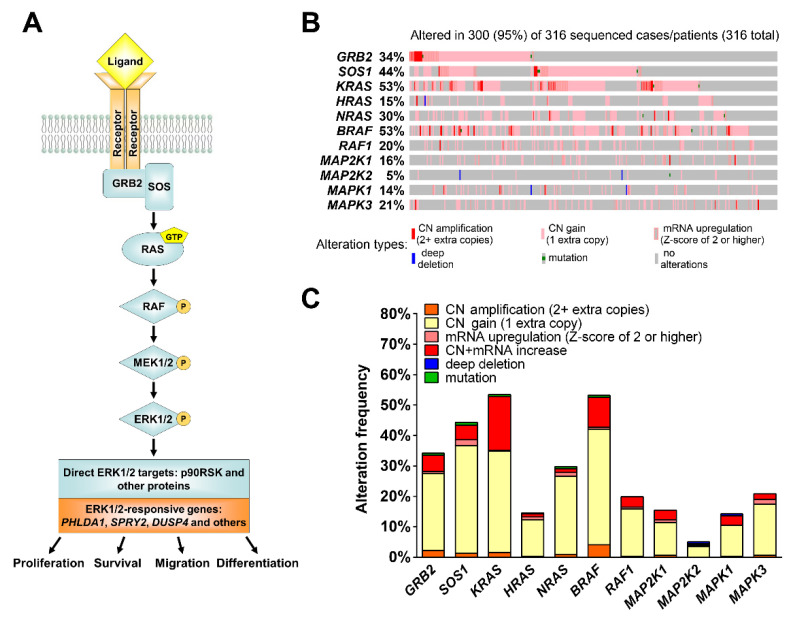
The MEK1/2 signaling pathway and its genetic alterations observed in HGSOC tissues. (**A**) The main elements of the MEK1/2 signaling pathway and cell properties controlled by its activity. (**B**) Genetic alterations and expression changes observed for MEK1/2 pathway elements in HGSOC tissues according to TCGA data. (**C**) Frequency of different genetic and transcriptional alterations of MEK1/2 pathway elements in HGSOC tissues. CN—copy number.

The MEK1/2 branch of the MAPK pathway mainly stimulates cell proliferation, migration, and differentiation (Figure 1A), while p38- and JNK/SAPK-associated signaling induces apoptosis, inflammation, and stress responses [31]. MEK1/2 signaling hyperactivation frequently occurs in malignant tumors and is therefore a promising target for anticancer therapy [32,33]. Because MEK1/2 selectively activates ERK1/2, its inhibition is an efficient way to suppress the activity of the whole cascade [34]. Based on clinical studies [35], MEK1/2 inhibitors received U.S. Food and Drug Administration (FDA) approval for tumors with activating BRAF mutations, including melanoma (trametinib, cobimetinib and binimetinib), non-small cell lung cancer (trametinib), and thyroid cancer (trametinib) (information available at https://www.fda.gov/ (accessed on 16 December 2020)).

The function of the MEK1/2-ERK1/2 pathway has been mainly studied in low-grade ovarian tumors due to high frequency of activating KRAS and BRAF mutations [36,37]. Despite the absence of KRAS/BRAF mutations in HGSOC, MEK1/2 signaling hyperactivation was reported in HGSOC and is associated with poor prognosis [38]. A limited amount of data suggests that MEK1/2-ERK1/2 pathway in HGSOC is responsible for development of cisplatin resistance [39,40]. The present paper aims to investigate the role of high MEK1/2 activity in the regulation of proliferation, viability, and stemness of HGSOC cells.

## 2. Materials and Methods

### 2.1. Clinical Tissue Samples

Data on MEK1/2 pathway genetic alterations were obtained from TCGA Research Network (https://www.cancer.gov/tcga, (accessed on 18 October 2018) database via cBioPortal [41,42]. Data on mutations, copy number alterations and mRNA expression for 316 complete samples from Ovarian Serous Cystadenocarcinoma (TCGA, Nature 2011) dataset were used.

Patient samples used for experiments were obtained in accordance with protocols approved by the University of Michigan’s IRB (HUM0009149). All participants provided a written consent for tumor collection. Tumors were processed for protein isolation as previously described [22]. Tissue microarray (TMA) slides constructed using paired clinical samples of HGSOC and normal fallopian tube tissue were commercially purchased from Lifespan Research Laboratories (Providence, RI, USA).

### 2.2. Cell Culture

OVCAR8, PEO4, and A2780 cells were provided by Dr. S. Murphy (Duke University, Durham, NC, USA). Kuramochi and OVSAHO cell lines were purchased from the Japanese Collection of Research Bioresources Cell Bank (Osaka, Japan). TOV21D (also known as TOV112D), HEY, and TOV21G cells were purchased from American Type Culture Collection (ATCC, Manassas, VA, USA), PEO1 cells were purchased from European Collection of Authenticated Cell Cultures (Millipore Sigma, Burlington, MA, USA). Mycoplasma was tested on a monthly base. OVCAR8, PEO4, Kuramochi, OVSAHO, and PEO1 are HGSOC cell lines, whereas A2780, TOV21D, HEY1, and TOV21G cells are Type I ovarian cancer. All cell lines were cultivated in RPMI-1640 medium (Corning, Tewksbury, MA, USA) supplemented with 5% FBS (Thermo Fisher Scientific, Waltham, MA, USA) and penicillin/streptomycin (Corning). PEO1 cells were cultivated in medium with the addition of 1 mM sodium pyruvate (Corning).

### 2.3. Drug Treatment of Cultured Cells

Compounds used included trametinib (Selleck Chemicals, Houston, TX, USA), Z-VAD-FMK (UBP Bio, Aurora, CO, USA), Nec-1 (ApexBio, Houston, TX, USA), cisplatin (ApexBio), and staurosporine (ApexBio). Trametinib, Z-VAD-FMK, Nec-1, and staurosporine were dissolved in DMSO (Fisher Scientific, Waltham, MA, USA) and cisplatin was dissolved in sterile water. Control samples in all experiments performed were treated with vehicle only. Vehicle concentration in growth medium did not exceed 0.2%. Cell treatment was performed by aspirating the growth media from the cells and replacing it with growth medium containing selected concentrations of drugs.

### 2.4. Immunoblotting

Total protein extracts were obtained from cell or tissue samples using Pierce RIPA buffer (Thermo Fisher Scientific, Waltham, MA, USA) according to manufacturer’s protocol. Protein concentrations were estimated using Pierce BCA Protein Assay Kit (Thermo Fisher Scientific). 40 μg of total protein were separated in Bolt 4–12% Bis-Tris Plus Gels (Thermo Fisher Scientific) and transferred to Hybond P 0.45 PVDF membranes (GE Healthcare, Chicago, IL, USA). Membranes were blocked with 5% BSA (Fisher Scientific) in tris-buffered saline with Tween-20 (TBST) (Fisher Scientific) and probed overnight at 4 °C with the following primary antibodies diluted in 5% BSA in TBST: pMEK1/2 (Cell Signaling Technology, Danvers, MA, USA, 41G9, 1:1000), pERK1/2 (Cell Signaling Technology, D13.14.4E, 1:1000), total ERK1/2 (Cell Signaling Technology, 137F5, 1:1000), pp90RSK1 (R&D Minneapolis, MN, USA, 1024A, 1:1000), GAPDH (ProteinTech, Rosemont, IL, USA, 1E6D9, 1:10,000), pSMAD2 (Cell Signaling Technology138D4, 1:1000), pSTAT3 (Cell Signaling Technology, D3A7, 1:1000), pGSK3b (Cell Signaling Technology, D85E12, 1:1000), pCRAF (Cell Signaling Technology, 56A6, 1:1000). Secondary antibodies were HRP-conjugated anti-rabbit IgG (Cell Signaling Technology, 7074, 1:10,000) or anti-mouse IgG (Cell Signaling Technology, 7076, 1:10,000) diluted in 5% skim milk (Millipore Sigma, Burlington, MA, USA) in TBST. Protein bands were developed using Luminata Classico or Luminata Forte HRP substrate (Millipore Sigma) and detected using Amersham Imager 600 (GE Healthcare, Chicago, IL, USA). After band detection, every membrane was incubated in Restore PLUS Western Blot Stripping Buffer (Thermo Fisher Scientific) for 30 min at room temperature and re-probed for GAPDH. Densitometric analysis of immunoblot images was performed using Image Lab V 6.1.0 software (Bio-Rad Laboratories, Hercules, CA, USA). Raw band intensity values obtained for proteins of interest were divided to corresponding intensity values of GAPDH bands detected using the same immunoblot membrane. Resulting values were additionally normalized to one of the samples, with relative band intensity level for this sample being equal to 1.00 (see Figure Legends for detailed description of each immunoblot experiment). All uncropped images of immunoblot membranes are available in the Supplementary Materials.

### 2.5. Immunohistochemical Staining of Tissue Slides

TMA slides were deparaffinized and rehydrated according to common protocols. Antigen retrieval was performed by boiling the slides in citrate buffer (pH = 6.0) for 10 min using a microwave oven. Sections were blocked using solution of 10% FBS (Thermo Fisher Scientific) and 1% BSA in TBS for 2 h at room temperature and probed with pERK1/2 antibodies (Cell Signaling Technology, D13.14.4E, 1:100 in TBS with 1% BSA) overnight at 4 °C. Slides were rinsed with 0.025% Triton-X100 (Fisher Scientific) in TBS, probed with secondary HRP-conjugated anti-rabbit IgG (Cell Signaling Technology, 7074, 1:1000 in TBS with 1% BSA) for 1 h at room temperature and developed with a 3,3-diaminobenzidine kit (Vector Laboratories, Burlingame, CA, USA). Separate slides were counterstained with hematoxylin (Fisher Scientific). After staining slides were rinsed with DI water, dehydrated and mounted using toluene (Fisher Scientific).

Tissue slides with tumor samples obtained from mice injected with PEO4 cells (see below) were stained at the histology core at the University of Michigan using EDTA-based antigen retrieval and mouse anti-ALDH antibody (BD Biosciences, San Jose, CA, USA, clone 44/ALDH; 1:100) as previously described [43]. For stain quantification, five sections from three tumors per treatment group were analyzed by two people. Counts were then compared using a 2-sided Student’s t test.

### 2.6. Immunofluorescent Staining of Tissue Slides

Tissue slides were deparaffinized and rehydrated according to common protocols. Antigen retrieval was performed by boiling the slides in citrate buffer (pH = 6.0) for 10 min using a microwave oven. Sections were blocked using solution of 10% FBS (Thermo Fisher Scientific) and 1% BSA in TBS for 2 h at room temperature and probed with pERK1/2 antibodies (Cell Signaling Technology, D13.14.4E, 1:100 in TBS with 1% BSA) overnight at 4 °C. Slides were rinsed with 0.025% Triton-X100 (Fisher Scientific) in TBS, probed with secondary Alexa488-conjugated anti-rabbit IgG (Cell Signaling Technology, 4412, 1:1000 in TBS with 1% BSA) for 1 h at room temperature and counterstained with 1 μg/mL 4′,6-diamidino-2-phenylindole (DAPI, Millipore Sigma). After staining slides were rinsed three times with TBS, coverslips were mounted using PermaFluor medium (Thermo Fisher Scientific).

### 2.7. RT-qPCR Analysis of Gene Expression

Total RNA was isolated from cell or tissue samples using TRIzol reagent and the PureLink RNA Mini Kit (Thermo Fisher Scientific) with additional on-column DNAse treatment. Reverse transcription was performed using the RevertAid RT Reverse Transcription Kit (Thermo Fisher Scientific). Quantitative PCR analysis of gene expression levels was performed in a CFX96 Touch thermocycler (Bio-Rad Laboratories) using PowerUp SYBR Green Master Mix (Thermo Fisher Scientific) and primers listed in Table S1. A three-step amplification program (15 s at 95 °C, 45 s at 62 °C, 30 s at 72 °C) was run for 40 cycles and reaction specificity was checked by melt curve analysis and agarose electrophoresis. Reaction efficiency was evaluated using standard curve approach and was within 98-102% for all primers. Transcript abundance was estimated using Pfaffl’s method [44], *TBP* was used as a housekeeping normalization gene.

### 2.8. Cell Viability Assay

Cells were plated in 12-well plates (Olympus Plastics, El Cajon, CA, USA) at 100,000 cells/well. After 24 h, growth medium was replaced with fresh medium containing compounds of interest. If Z-VAD-FMK or Nec-1 were used in treatment, cells were pre-treated with aforementioned compounds for 45 min before adding other compounds. After 72 h of treatment, cells were harvested by trypsinization, pelleted, and resuspended in PBS. Numbers of viable and dead cells were assessed by direct counting using a Countess automated cell counter (Thermo Fisher Scientific) in the presence of 0.4% Trypan Blue. IC50 values were estimated based on relative viable cell numbers obtained for cells treated with different concentrations of cisplatin or trametinib.

### 2.9. Real-Time Cytotoxicity Assay

Cells were plated in 96-well plates (Olympus Plastics) at 5000 cells/well. After 24 h, growth medium was replaced with fresh medium containing compounds of interest and Cytotox Green Reagent (Essen BioScience, Ann Arbor, MI, USA). Dead cells were detected in real time for 72 h using the IncuCyte S3 cell imaging system (Essen BioScience). The relative cytotoxicity level was estimated as the number of green fluorescent objects normalized to corresponding cell confluence values. Staurosporine (200 nM) was used as a positive control to induce cell death.

### 2.10. Average Cell Size Estimation

Cells were plated in 96-well plates (Olympus Plastics) at 5000 cells/well. After 24 h, growth medium was replaced with fresh medium containing compounds of interest. After 72 h of treatment, plates with the cells were imaged us-ing IncuCyte S3 system (Essen BioScience). The total area covered with cells was estimated using ImageJ2 software [45] and divided by total number of cell nuclei in the analyzed image.

### 2.11. Cell Cycle Assay

Cells were plated in 12-well plates at 100,000 cells/well. After 24 h, growth medium was replaced with fresh medium containing compounds of interest. After 24 h of treatment, cells were harvested by trypsinization, resuspended in 300 μL of ice-cold PBS, and fixed by the addition of 0.7 mL of ice-cold 70% ethanol in a dropwise manner with constant mixing. After addition of ethanol, samples were stored at −70 °C overnight. Fixed cell samples were washed with ice-cold ethanol twice, treated with 0.2 mg/mL RNAse A for 60 min at 37 °C, and stained with 10 µg/mL propidium iodide. Stained samples were analyzed using the FACSCalibur flow cytometer (BD Biosciences) and ModFit LT software (Verity Software House, Topsham, ME, USA), at least 25,000 qualifying events were detected in each evaluated sample.

### 2.12. Knockdown of Gene Expression with siRNA

Cells were plated in 12-well plates at 50,000 cells/well. After 24 h, growth medium was replaced with fresh medium, and cells were transfected with 30 pmol ON-TARGETplus control non-targeting siRNA or SMARTpool siRNA against human RIPK1 (Dharmacon, Lafayette, CO, USA) using Lipofectamine RNAiMAX reagent (Thermo Fisher Scientific) according to manufacturer’s protocol. Cells were transfected overnight, and the transfection medium was replaced with fresh medium next morning. After 6 h, cells were treated with drugs as described above.

### 2.13. Estimation of CD133+ Cell Fraction

Cells were harvested by trypsinization, washed with cold PBS, resuspended in PBS and stained with anti-CD133 antibodies conjugated with APC fluorophore (Miltenyi Biotec, Bergisch Gladbach, Germany, 293C3, 1:50) for 30 min at 4 °C. Stained cells were washed with cold PBS and resuspended in PBS containing 0.1 μg/mL DAPI to exclude dead cells from analysis. Samples were analyzed using CytoFLEX flow cytometer and CytExpert software (Beckman Coulter, Brea, CA, USA) according to previously described algorithms [22].

### 2.14. Estimation of ALDH Activity

Cells were harvested by trypsinization, washed with cold PBS and stained using the ALDEFLUOR Kit (STEMCELL Technologies, Vancouver, BC, Canada) according to a previously described protocol [22]. Stained cells were resuspended in ALDEFLUOR Assay Buffer containing 0.1 µg/mL DAPI to exclude dead cells from analysis. Samples were analyzed using the CytoFLEX flow cytometer and CytExpert software (Beckman Coulter). Cells displaying ALDEFLUOR signal at least 10-fold higher than median values were considered “high-positive” and their fraction was evaluated separately.

### 2.15. Spheroid Cell Growth Assay

Cells were seeded in 24-well ultra-low attachment plates (Corning) at 2000 cells/well. After 7 days, cell clusters were harvested and disrupted by mild trypsinization, pelleted, and resuspended in PBS. Numbers of viable and dead cells were assessed by direct counting using a Countess automated cell counter (Thermo Fisher Scientific) in the presence of 0.4% Trypan Blue.

### 2.16. Animal Studies

All experiments were performed with approval of the University Committee on Use and Care of Animals at the University of Michigan (Ann Arbor, MI, USA). PEO-4 cells (100,000) in 100 μL of Matrigel (BD Biosciences) were injected subcutaneously into the axillae of 8-week-old female NOD.Cg-Prkdcscid Il2rgtm1Wjl/SzJ (NSG) mice. Mice were randomly assigned to one of the two treatment conditions. Three days after cell injection, the mice were treated with IP injections of DMSO or 1 mg/kg trametinib daily (*n* = 10 mice per treatment group) for 25 days. This is based on a final tumor volume of control animals of ~1000 mm^3^ with an expected standard deviation of 30%. The experiments proposed will have 82.5% power to detect a 35% reduction in tumor. Tumors were measured using calipers, and tumor volume (L × W × W/2) was calculated by two scientists who were not part of treatment group. After 25 days, mice were sacrificed, and tumor tissue samples were collected for analysis.

### 2.17. Statistical Analysis

At least 3 independent biological replicates were performed for each cell culture experiment, the exact sample sizes are provided in corresponding figure legends. Gene expression levels were considered to be log-normally distributed [46] and differences between sample groups were evaluated using two-tailed Student’s T-test with Welch’s correction for unequal variances after logarithmic transformation of data. Differences between sample groups in cell culture experiments and in vivo experiments were evaluated using two-tailed Mann-Whitney U-test (data distribution normality for sample sizes higher than *n* = 7 was rejected based on Shapiro-Wilk test) unless it is stated different in the figure legend. Statistical significance was accepted with *p* < 0.05. Asterisks “*”, “**”, and “***” denote *p* < 0.05, *p* < 0.01, and *p* < 0.001, respectively. Statistical analysis was performed in OriginPro 2016 software (OriginLab Corporation, Northampton, MA, USA). Half-maximal inhibitory concentration (IC50) and 50% growth inhibitory concentration (GI50) values were calculated using the nonlinear regression algorithm in Prism 7 software (GraphPad Software, San Diego, CA, USA). Comparison of IC50 and GI50 values for different cell lines treated in the same way was performed based on overlap of 95% confidence intervals.

## 3. Results

### 3.1. The MEK1/2 Pathway Is Active in High Grade Ovarian Tumors

Activation of MEK1/2 signaling frequently occurs in cancer cells and promotes cell proliferation [32]. The MEK1/2 pathway has a clear hierarchical structure of signal transduction (Figure 1A, Table S2). We used data available from The Cancer Genome Atlas (TCGA) Research Network database (https://www.cancer.gov/tcga (accessed on 18 October 2018)) to analyze genetic and transcriptional changes in MEK1/2 pathway components occurring in HGSOC [41,42]. While *KRAS* and *BRAF* mutations only occur in 1.26% of HGSOC cases, 25% of cases display amplifications (2 or more extra copies) in one or more of genes involved in MAPK signal transduction (Table S2). Taking local genetic gains (1 extra copy) and mRNA overexpression events into account increases the percentage of cases with pro-active changes in the MEK1/2 pathway to 95% (Figure 1B). *SOS1*, *KRAS*, and *BRAF* are more often affected by these alterations compared to their downstream targets, MEK and ERK (Figure 1C). Deletions of MEK-related genes are very rare in HGSOC comprising 1.6% of all cases (Figure 1B).

**Figure 2:**
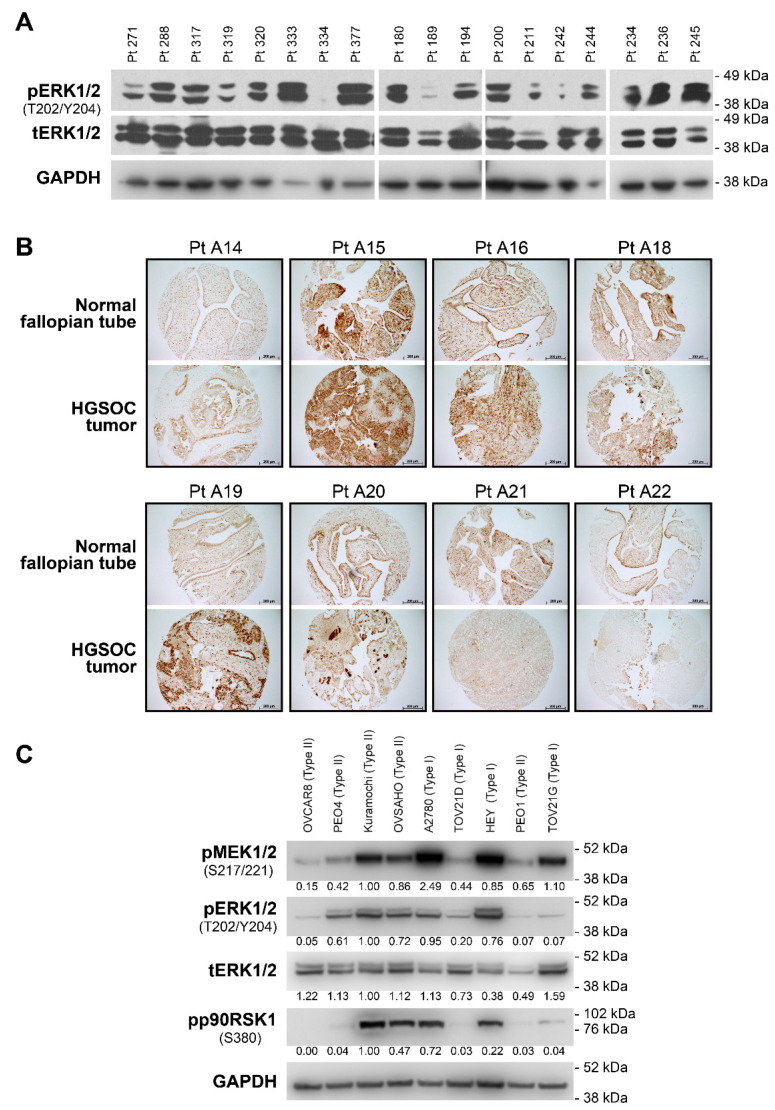
(**A**) Immunoblotting analysis of phosphorylated and total ERK1/2 levels in representative set of 18 clinical samples of HGSOC tissue. (**B**) Immunohistochemical staining of phosphorylated ERK1/2 in 8 representative paired clinical samples of HGSOC and normal fallopian tube tissues. Scale bars: 200 μm. (**C**) Immunoblotting analysis of MEK1/2-ERK1/2 pathway component activities in various ovarian cancer cell lines. Numbers under the bands represent relative intensity normalized to GAPDH level and Kuramochi sample used as a positive control for all proteins. pERK1/2—phosphorylated ERK1/2, tERK1/2—total ERK1/2, pMEK1/2—phosphorylated MEK1/2, pp90RSK1—phosphorylated p90RSK1, Pt XXX—patient number XXX as provided by Lifespan Research Laboratories.

To confirm activity at the protein level, we next analyzed the level of phosphorylated and total ERK1/2 (pERK1/2 and tERK1/2, respectively) in 43 clinically obtained HGSOC tumors. We detected pERK1/2 bands in 84% (36 of 43 cases) of samples using immunoblotting (see Figure 2A for representative set of 18 samples and Supplementary Immunoblotting Data for original immunoblot images for all examined samples). This observation was further confirmed using TMA slides constructed from paired samples of HGSOC and benign fallopian tube obtained from 10 independent patient samples. IHC analysis detected positive pERK1/2 staining in nine out of 10 tumor samples with staining intensity being similar or more prominent than in the corresponding normal samples (Figure 2B and Figures S1–S3). The MEK1/2 pathway is also hyperactive in various ovarian cancer cell lines as indicated by high pMEK1/2, pERK1/2 and pp90RSK1 levels (Figure 2C).

**Figure 3:**
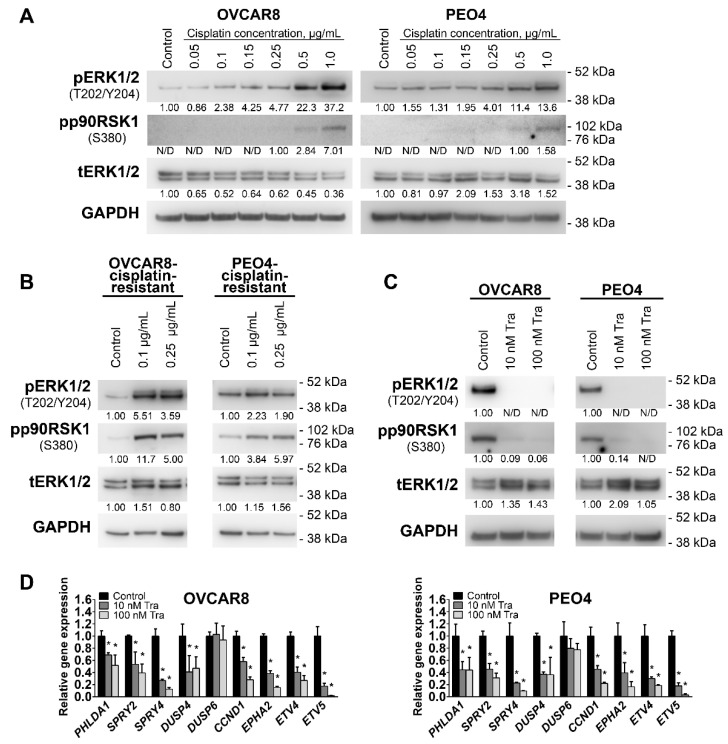
(**A**) Immunoblotting analysis of MEK1/2-ERK1/2 pathway component activities in cells treated with cisplatin for 24 h. Numbers under the bands represent relative intensity normalized to GAPDH levels and Control samples, except pp90RSK1 data that were normalized to samples showing the weakest detectable band intensity. (**B**) Immunoblotting analysis of MEK1/2-ERK1/2 pathway component activities in cells resistant to the indicated concentrations of cisplatin. Numbers under the bands represent relative intensity normalized to GAPDH levels and Control samples. (**C**) Immunoblotting analysis of MEK1/2-ERK1/2 pathway component activation in cells treated with trametinib for 24 h. Numbers under the bands represent relative intensity normalized to GAPDH levels and Control samples. (**D**) Gene expression levels of MEK1/2-responsive genes in cells treated with trametinib for 10 h. Data are normalized to “Control” samples and presented as mean+S.D. (*n* = 3, two-tailed Student’s T-test with Welch’s correction, * - *p* < 0.05). Tra—trametinib, pERK1/2—phosphorylated ERK1/2, tERK1/2—total ERK1/2, pp90RSK1—phosphorylated p90RSK1, N/D—non-detectable signal.

Activation of MEK1/2 pathway was previously reported in some HGSOC cells in response to high doses of cisplatin and may be linked to development of cisplatin resistance [39,40,47,48]. We therefore used OVCAR8 and PEO4 cell lines (displaying moderate pMEK1/2 levels, Figure 2C) to confirm that these effects are reproduced in our model systems. While both OVCAR8 and PEO4 cells are already considered platinum-resistant [49,50,51], we sought to evaluate the possible effects of repeated platinum-based treatment. As expected, cisplatin treatment of OVCAR8 and PEO4 cells (Figure S4A) enhanced ERK1/2 and p90RSK1 activation in response to increasing doses of drug (Figure 3A). We therefore generated OVCAR8 and PEO4 cell cultures with even higher cisplatin resistance by prolonged cultivation of cells in the presence of 0.1 μg/mL or 0.25 μg/mL of cisplatin. In agreement with data reported above, development of cisplatin resistance was associated with enhanced ERK1/2 and p90RSK1 activity (Figure 3B). To additionally confirm MEK1/2-ERK1/2 signaling changes, we evaluated expression of 10 genes (*PHLDA1*, *SPRY2*, *SPRY4*, *DUSP4*, *DUSP6*, *CCND1*, *EPHA2*, *EPHA4*, *ETV4*, *ETV5*, see Table S3 for details) that were reported to reflect MEK1/2 pathway activity [52]. We detected at least a two-fold increase in *PHLDA1*, *SPRY2*, *DUSP4*, *DUSP6*, and *EPHA2* expression in cisplatin-resistant cells (Figure S4B). *EPHA4* expression levels in examined cells were below the detection limit, so this gene was excluded from this and subsequent gene expression analyses. These results, taken together with previously published observations, suggest an important role of high MEK1/2-ERK1/2 activity in HGSOC development of chemoresistance.

### 3.2. Inhibition of MEK1/2 Causes Arrest of HGSOC Cell Proliferation without Inducing Cell Death

We next evaluated the impact of a selective and non-competitive FDA approved inhibitor MEK1/2 inhibitor, trametinib [35], on HGSOC proliferation. Activity of the MEK1/2 pathway was completely inhibited by 10 nM or higher concentrations of trametinib after 24 h of treatment. Furthermore, trametinib-induced inactivation of the MEK1/2-ERK1/2 cascade caused corresponding decreases in the level of pp90RSK1, a downstream ERK1/2 target (Figure 3C). MEK1/2 inhibition was also reflected in substantial dose-dependent downregulation of 8 out of 9 MEK-responsive genes expressed in OVCAR8 and PEO4 cells after treatment with trametinib for 10 h (Figure 3D). These results indicate that trametinib is a potent inhibitor of MEK1/2 signaling activity in HGSOC cells that downregulates the activity of the entire MEK1/2-ERK1/2 axis, including downstream targets. Cisplatin-resistant OVCAR8 and PEO4 cells display increased resistance to MEK1/2-ERK1/2-inhibiting action of cisplatin indicated by retention of detectable pERK1/2 levels in cells treated with 10 nM trametinib (Figure S5). However, this effect is compensated by higher sensitivity to physiological trametinib impact (see below, Figure S6D).

**Figure 4:**
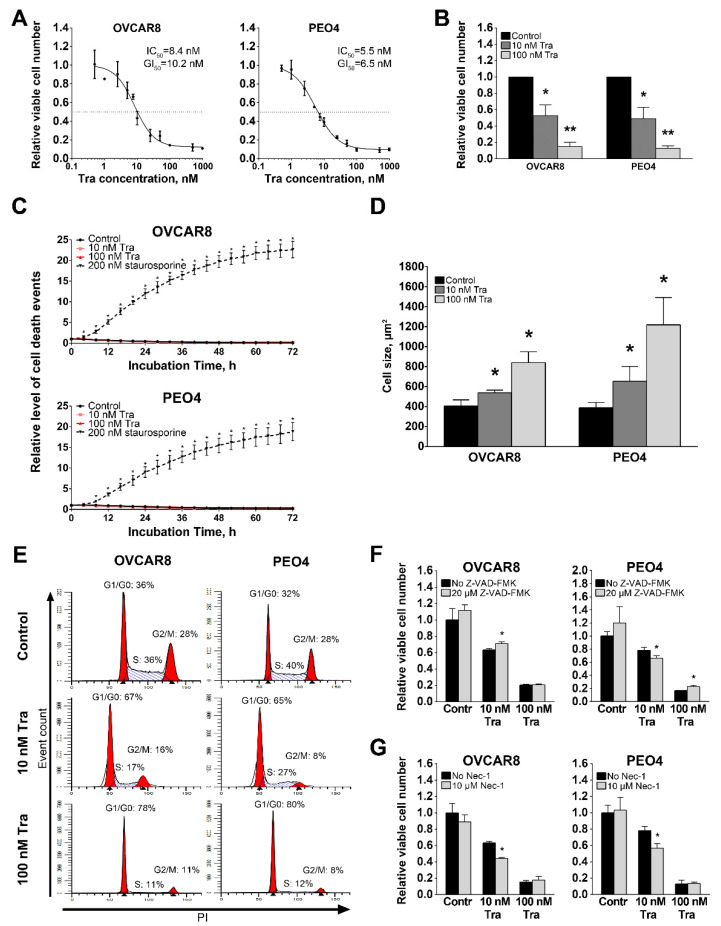
Changes in functional characteristics of HGSOC cell cultures caused by trametinib treatment. (**A**) Dose-response curves generated using relative viable cell numbers after treatment with various concentrations of trametinib for 72 h. Data are normalized to vehicle-treated control samples (not shown) and presented as mean±S.D. (*n* = 3). (**B**) Viable cell numbers after treatment with selected concentrations of trametinib for 72 h. Data are normalized to “Control” samples and presented as mean + S.D. (*n* = 6, Mann-Whitney U-test, * - *p* < 0.05, ** - *p* < 0.01). (**C**) Cytotoxic effect of trametinib treatment detected using the CytoTox reagent. Staurosporin is used as a positive control. Fluorescence level for each time point is normalized to the area covered by cells and starting value; data are presented as mean ± S.D. (*n* = 4, Mann-Whitney U-test, * - *p* < 0.05). (**D**) Average cell size after treatment with trametinib for 72 h. Data are presented as mean + S.D. (*n* = 4, Mann-Whitney U-test, * - *p* < 0.05). (**E**) Cell cycle phase analysis after trametinib treatment for 24 h. (**F**) Effect of pan-caspase inhibitor Z-VAD-FMK on cells treated with trametinib for 72 h. Data are normalized to “Control, No Z-VAD-FMK” sample and presented as mean + S.D. (*n* = 4, Mann-Whitney U-test, * - *p* < 0.05). (**G**) Effect of the RIPK1 inhibitor necrostatin-1 on cells treated with trametinib for 72 h. Data are normalized to “Control, No Nec-1” sample and presented as mean + S.D. (*n* = 6, Mann-Whitney U-test, * - *p* < 0.05). Tra—trametinib, PI—propidium iodide, Nec-1—necrostatin-1.

To evaluate the role of MEK1/2 activity on HGSOC proliferation, we treated cells for 72 h with a wide range of trametinib concentrations (0.5 nM–1000 nM). The resulting IC50 and GI50 values were 8.4 nM and 10.2 nM for OVCAR8 cells and 5.5 nM and 6.5 nM for PEO4 cells, respectively (Figure 4A), with a maximum effect at 100 nM. Based on these results, two trametinib concentrations (10 nM and 100 nM) were chosen for further experiments. Treatment with 100 nM trametinib reduced viable cell numbers to 15% (OVCAR8) and 13% (PEO4) of control values (Figure 4B) without causing considerable cytotoxic action (Figure 4C and Figure S6A,B). Imaging of live cells treated with trametinib revealed significant increase in cell size (Figure 4D and Figure S6C). Cisplatin-resistant OVCAR8 and PEO4 cells displayed higher sensitivity to cytostatic but not cytotoxic effects of trametinib treatment (Figure S6D,E). Cell cycle analysis demonstrated that trametinib treatment caused arrest of proliferation in the G1/0-phase but did not affect the sub-G0 fraction of apoptotic cells (Figure 4E). Enrichment of G1/0-phase cells may partially explain the increase in cell size, since these cells accumulate proteins, nucleic acids and nutrients required for DNA replication in S-phase.

While caspase inhibition with Z-VAD-FMK attenuated apoptosis induction by 0.2 µM staurosporine (positive control, Figure S7A), it did not cause prominent impact trametinib-induced changes in viable cell numbers (Figure 4F). Similarly, inhibition of the major necroptosis regulator RIPK1 [53] with 10 µM Nec-1 (Figure 4G) or siRNA-mediated knockdown of RIPK1 expression (Figure S7B,C) did not attenuate trametinib-induced effects. On the opposite, combination of Nec-1 and 10 nM trametinib resulted in further suppression of cell proliferation. These results suggest that the reduction in cell number is not due to either caspase-dependent apoptosis or RIPK1-dependent necroptosis.

### 3.3. Trametinib Promotes Stemness of HGSOC Cells

**Figure 5:**
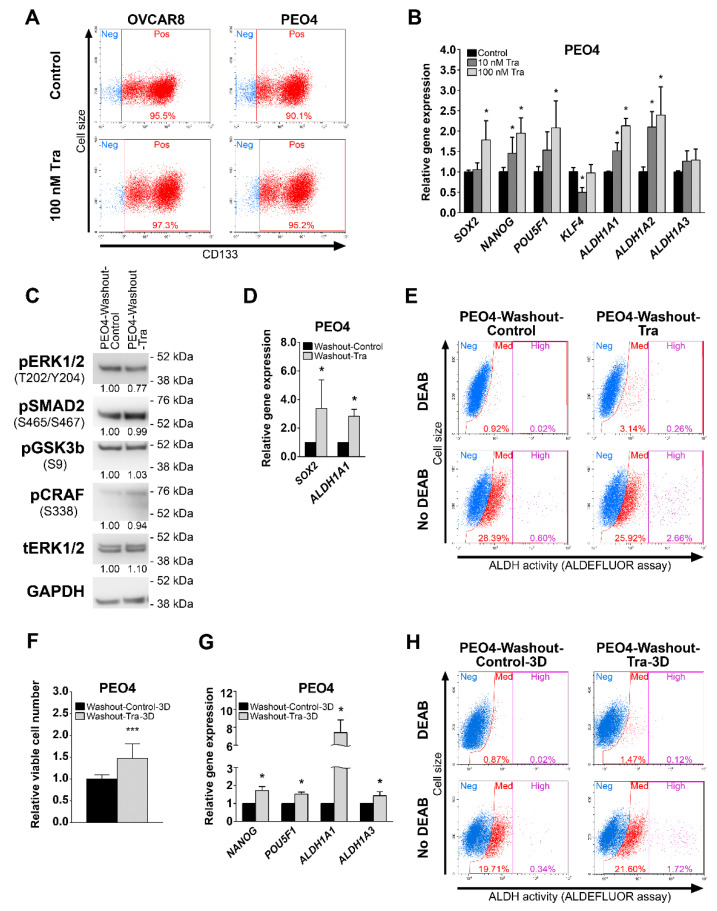
Effects of trametinib treatment on stemness-related characteristics of HGSOC cells. (**A**) Expression of the CD133 surface marker in cells treated with trametinib for 72 h. Gates indicate CD133-negative (“Neg”) and CD133-positive (“Pos”) cell subpopulations. (**B**) Gene expression levels of stemness-related genes in cells treated with trametinib for 10 h. Data are normalized to “Control” samples and presented as mean + S.D. (*n* = 3, two-tailed Student’s T-test with Welch’s correction, * - *p* < 0.05). (**C**) Immunoblotting analysis of various signaling proteins activation in PEO4-Washout cells. Numbers under the bands represent relative intensity normalized to GAPDH levels and PEO4-Washout-Control sample. (**D**) Gene expression levels of stemness-related genes in PEO4-Washout cells. Data are normalized to “Washout-Control” samples and presented as mean + S.D. (*n* = 3, two-tailed Student’s T-test with Welch’s correction, * - *p* < 0.05). (**E**) ALDEFLUOR analysis of PEO4-Washout cells. *N*,*N*-diethylaminobenzaldehyde (DEAB) was used as ALDH inhibitor to define background ALDEFLUOR signal and set proper gates for ALDH-positive cells. Gates indicate cell subpopulations displaying negative (“Neg”), medium (“Med”), or high (“High”) levels of ALDH activity. (**F**) Viable cell numbers of PEO4-Washout cells after cultivation in non-adherent 3D conditions for 7 days. Data are normalized to “Washout-Control-3D” samples and presented as mean + S.D. (*n* = 9, Mann-Whitney U-test, *** - *p* < 0.001). (**G**) Gene expression levels of stemness-related genes in PEO4-Washout cells after cultivation in non-adherent 3D conditions for 7 days. Data are normalized to “Washout-Control-3D” samples and presented as mean + S.D. (*n* = 3, two-tailed Student’s T-test with Welch’s correction, * - *p* < 0.05). (**H**) ALDEFLUOR analysis of PEO4-Washout cells after cultivation in non-adherent 3D conditions for 7 days. DEAB was used as ALDH inhibitor to define background ALDEFLUOR signal and set proper gates for ALDH-positive cells. Gates indicate cell subpopulations displaying negative (“Neg”), medium (“Med”), or high (“High”) levels of ALDH activity. Tra—trametinib.

CSCs are associated with chemoresistance and recurrence [17,18,22,23], so we investigated the effect of trametinib on cancer stemness. Both OVCAR8 and PEO4 cell lines display high percentages of CD133+ cells that are not reduced by trametinib treatment (Figure 5A). In contrast, trametinib treatment of PEO4 cells for 10 h significantly promoted the expression of cell stemness regulators *SOX2*, *NANOG*, *POU5F1* (*OCT4*), and two *ALDH1A* homologs associated with elevated chemoresistance and the stem-like phenotype of ovarian cancer cells [18,22,54,55] (Figure 5B). We next treated PEO4 and OVCAR8 cells with vehicle or 100 nM trametinib for 72 h, then performed drug washout to propagate the cells selected by the treatment (hereinafter denoted as “PEO4-Washout-Control” and “PEO4-Washout-Tra”, respectively, Figure S8A, or “OVCAR8-Washout-Control” and “OVCAR8-Washout-Tra”, respectively, Figure S9A). PEO4-Washout-Tra cells displayed no prominent differences in activity of MEK1/2 or other signaling pathways (Figure 5C) or expression levels of MEK1/2-responsive genes (Figure S8B) in comparison to control cells. Trametinib-driven selection had no biologically relevant effect upon cell proliferation rate (Figure S8C), sensitivity to cisplatin (Figure S8D), or percentage of CD133+ cells (Figure S8E); but resulted in *SOX2* and *ALDHA1A* expression upregulation in comparison to control cells (Figure 5D and Figure S8F). PEO4-Washout-Tra also displayed enrichment of cells with very high ALDH activity (Figure 5E, “High” gate). Similar effects were observed in OVCAR8 cells (Figure S9), but no increase in *ALDH1A1* expression (Figure S9F,G) was determined in cells growing in 2D.

The ability to grow in non-adherent conditions is a distinct feature of CSCs. We therefore conducted a spheroid formation assay and detected a significantly higher growth rate of both PEO4-Washout-Tra and OVCAR8-Washout-Tra cells in comparison to control (Figure 5F and Figure S10A). Moreover, induction of *ALDH1A1* expression observed in adherent PEO4-Washout-Tra cells was further increased in spheroids and was accompanied by upregulation of *NANOG* and *POU5F1* expression (Figure 5G and Figure S8H). PEO4-Washout-Tra cells grown as spheroids retained a very high percentage of CD133+ cells (>90%, Figure S8G) and a higher fraction of ALDEFLUOR-bright cells compared to control cells (Figure 5H, “High” gate). OVCAR8-Washout-Tra cells grown in non-adherent conditions demonstrated similar trends in gene expression and fraction of CD133+ cells (Figure S10B,C), while the subpopulation of ALDH-positive cells prominently increased (Figure S10D). Taken together, these results indicate that cells surviving trametinib treatment obtain a more pronounced CSC phenotype.

### 3.4. Effect of MEK1/2 Inhibition In Vivo

**Figure 6:**
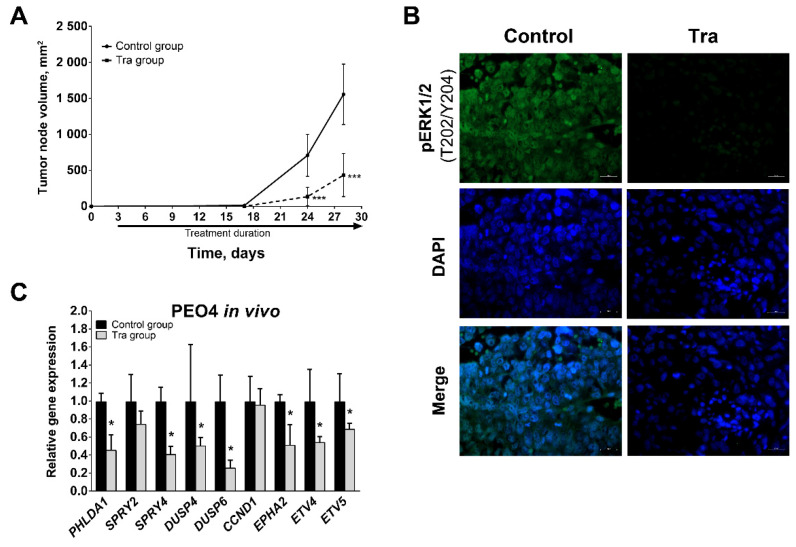
Effects of trametinib treatment on HGSOC growth in vivo. (**A**) Growth kinetics of tumors developed from subcutaneously injected PEO4 cells (*n* = 10, Mann-Whitney U-test, *** - *p* < 0.001). (**B**) Immunofluorescent staining of phosphorylated ERK1/2 (green) in PEO4 xenograft tissue samples. Cell nuclei were counterstained with DAPI (blue). Scale bars: 20 μm. (**C**) Gene expression levels of MEK1/2-responsive genes in PEO4 xenograft tissue samples. Data are normalized to “Control group” samples and presented as mean + S.D. (*n* = 3, two-tailed Student’s T-test with Welch’s correction, * - *p* < 0.05). Tra—trametinib, pERK1/2—phosphorylated ERK1/2.

To examine the impact of MEK1/2 inhibition in vivo, we injected PEO4 cells subcutaneously into mice that were subsequently treated intraperitoneally with 1 mg/kg trametinib or vehicle daily. Trametinib treatment significantly reduced the rate of tumor growth and caused a 4-fold decrease in tumor volume as estimated at 4 weeks after initial cell injection (Figure 6A). Xenograft tumors grown in mice from both control and trametinib-treated experimental groups displayed tissue morphology typical of HGSOC (Figure S11). Immunofluorescent staining revealed drastic decrease of pERK1/2 levels in xenograft tissue samples obtained from trametinib-treated mice (Figure 6B, for high-resolution images see Figure S12A); this result was additionally confirmed by immunoblotting analysis (Figure S12B). Inhibition of MEK1/2-ERK1/2 activity was further confirmed by RT-qPCR analysis that revealed the reduction of *PHLDA1*, *SPRY4*, *DUSP4*, *DUSP6*, *EPHA2*, *ETV4*, and *ETV5* expression levels (Figure 6C). In contrast with the changes observed in cells, *SPRY2* and *CCND1* expression was not affected by trametinib in vivo. Xenografts from trametinib-treated mice displayed increased ALDH1 levels assessed via immunohistochemical staining in comparison to samples obtained from control group (Figure S13), suggesting that trametinib treatment caused CSC enrichment in tumor tissue. Thus, trametinib treatment of tumors in vivo caused prominent inhibition of MEK1/2 pathway activity, reduced the rate of tumor growth, and promoted stem-like characteristics of tumor cells in full concordance with results obtained in experiments in vitro.

## 4. Discussion

The present study evaluated the role of the MEK1/2 pathway in HGSOC and assessed MEK1/2 inhibition as a therapeutic approach. Activation of the MAPK signaling pathway is one of the most frequent events in cancer and affects many features inherent to malignant cells [32,56]. Most notably, high activity of the MEK1/2-ERK1/2 portion of the MAPK cascade directly promotes cell proliferation, survival, and drug resistance [31,32,33,57]. Furthermore, a number of studies reported that ERK1/2 activation occurs in CSCs [27] and is crucial for cell survival and proliferation in prostate, breast, and thyroid tumors [28,29,30]. A special focus in MAPK signaling inhibition is placed on MEK1/2 as it acts as a “gatekeeper” of MAPK pathway, conducting the signal from multiple upstream regulators towards ERK1/2, which are the only downstream targets of MEK1/2 [34]. Development of a new generation of non-competitive, highly specific MEK1/2 inhibitors (trametinib [58], selumetinib [59], cobimetinib [60], and others) that show high efficiency and tolerable side effects significantly increases the therapeutic potential of MEK1/2 inhibition.

Despite a lack of mutations activating KRAS or BRAF in HGSOCs, we observed high levels of MEK1/2-ERK1/2 activity in a majority of clinical samples and cell lines. This observation is further supported by recently published studies reporting an association between high MAPK activity in HGSOC and poor survival [38,61]. Moreover, cisplatin treatment of HGSOC cells results in further MEK1/2 pathway activation that persists in cisplatin-resistant cells. MEK1/2-ERK1/2 signaling inhibition in ovarian cancer cells is reported to sensitize them to chemotherapy [57], so MEK1/2 pathway hyperactivation could be a mechanism allowing HGSOC cells to overcome cytotoxic effects of cisplatin. Cisplatin-induced MEK1/2 activation in OVCAR8 and PEO4 cell lines established from recurrent tumors, which already obtained cisplatin resistance [49,50,51], suggests that repeated cisplatin treatment can further promote MEK1/2-ERK1/2 activity and associated chemoresistance in HGSOC. Such an effect could possibly be facilitated through cisplatin-driven enrichment of chemoresistant CSCs that often display high MEK1/2 pathway activity.

TCGA data suggest that MEK1/2 signaling hyperactivation is caused by genetic amplifications and overexpression of upstream MAPK components (GRB2, SOS1, RAS, and RAF families) and therefore could be countered by MEK1/2 inhibition. Indeed, treatment of ovarian cancer cell lines with the MEK1/2 inhibitor, selumetinib, results in prominent inhibition of ERK1/2 phosphorylation [61]. Considering the MEK1/2-activating effects of cisplatin discussed above, we focused our studies on cisplatin-resistant cells, OVCAR8 and PEO4. These cell lines also display high ALDH1 activity and prominent subpopulations of CD133+ cells, two distinct characteristics of ovarian CSCs [22,54,62,63,64,65].

Trametinib treatment drastically reduces the growth rates of HGSOC cells both in culture and in vivo, confirming that MEK1/2-ERK1/2 activity is involved in driving cell proliferation [31]. Anti-proliferative effects of MEK1/2 inhibitors have been reported in many cell types, including ovarian cancer cells, and cause G1/0-phase cell cycle arrest due to loss of ERK1/2 activation [38,61,66,67]. However, despite pronounced cytostatic action in vitro, most first generation MEK1/2 inhibitors demonstrated limited efficacy in clinical trials involving melanoma, breast, colon, lung, and pancreatic cancer patients [35,68]. Trametinib, on the other hand, belongs to a new generation of MEK1/2 inhibitors, displays higher efficiency, and was the first MEK1/2 inhibitor approved by FDA for cancer treatment [35]. Thus, trametinib could possibly show greater efficacy in clinical trials. This suggestion is supported by a recent case report describing partial response to trametinib treatment in a recurrent HGSOC patient with an extensive history of previous treatments, including carboplatin, paclitaxel, bevacizumab, gemcitabine, and doxorubicin [69]. Trametinib treatment resulted in reduction of tumor nodules’ size and serum CA125 levels with tolerable adverse effects. It is, however, noteworthy that this particular HGSOC case had an activating KRAS mutation that warranted for MEK1/2 inhibitor treatment attempt; nevertheless, similar effects of MEK1/2 inhibitors may be expected if MEK1/2-ERK1/2 pathway activity is elevated in tumors with wild-type KRAS.

We observed that trametinib induces cell cycle arrest in HGSOC lines. The potential of cell cycle arrest in HGSOC is best illustrated by paclitaxel that blocks cell cycle in the G2/M-phase. Prolonged mitotic block results in apoptosis induction and eventual cell death [70,71]. In a similar way, inhibition of MEK1/2 with trametinib induced death of various tumor cells that are heavily dependent on elevated RAS-RAF-MEK1/2-ERK1/2 cascade activity [72,73,74,75]. In contrast to these reports, we did not detect any cytotoxic effects caused by MEK1/2 inhibition in HGSOC cells. Recently another MEK1/2 inhibitor, selumetinib, was reported to induce apoptosis in the PEO1 HGSOC cell line [61]. This discrepancy can be explained by the fact that both OVCAR8 and PEO4 cells represent cisplatin-resistant subgroups of HGSOC in comparison to cisplatin-sensitive PEO1 cells and therefore may have developed various means of avoiding cell death. Our results indicate that active MEK1/2 signaling, while promoting proliferation of cisplatin-resistant HGSOC cells, is not essential for their survival. Moreover, loss of MEK1/2-regulated negative feedback can induce RAF hyperactivation that promotes cell viability through MEK1/2-unrelated pathways [68].

Cells surviving trametinib treatment obtain a prominent stem-like phenotype, including increased ability to grow under non-adherent conditions, increased ALDH1 activity, and a very high CD133+ fraction typical of ovarian CSCs [65]. This effect could be due to trametinib favoring the propagation of pre-existing CSC subpopulations or directly inducing stem-like properties in affected cells. Our results indicate that the latter option is more likely, as trametinib increased expression of stemness-related genes after only 10 h of treatment. Furthermore, trametinib-driven cell stemness was persistent for at least 10 passages after drug washout despite MEK1/2-ERK1/2 activity being restored to control levels. However, the differences in changes of stemness-related genes expression observed immediately after trametinib treatment (Figure 5B), in adherent “PEO4-Washout-Tra” cells (Figure 5D) and in “PEO4-Washout-Tra” cells grown in suspension (Figure 5G) suggest that the MEK1/2-ERK1/2 pathway may control cell stemness indirectly, with other mechanisms having significant impact upon the resulting stem-like cell phenotype.

An additional study previously reported anti-stemness impact of MEK1/2 inhibition in PEO1 cells [61], suggesting differences between cisplatin-sensitive and cisplatin-resistant HGSOC cells. Similar stemness-promoting effect of MEK1/2 inhibitors was observed in colorectal cancer due to Wnt/β-catenin pathway activation [76]. Also, ALDH1 overexpression in response to BRAF and MEK1/2 inhibition was recently reported in melanoma [77]. Given that high expression of ALDH1 and CD133 in ovarian tumors is strongly associated with poor prognosis and chemoresistance [78,79,80,81,82], we conclude that prolonged treatment of cisplatin-resistant HGSOC with trametinib might promote CSC enrichment. Since CSCs are currently considered as a major source of tumor recurrence [18,25,26], MEK1/2-related promotion of cancer cell stemness could possibly underlie a failure of several clinical trials focused on trametinib alone or as a part of complex anti-tumor therapy [83,84].

## 5. Conclusions

The present study demonstrates that treatment with trametinib as a single drug delays HGSOC tumor growth and therefore has the potential for prolonging disease-free survival of HGSOC patients. Because cisplatin-resistant cells are more sensitive to trametinib, this drug may prove to be an important therapeutic option for platinum-resistant recurrent tumors and initially cisplatin-refractory cases. Due to its fewer side effects, trametinib treatment can also be considered for patients who are unable to tolerate certain chemotherapeutic regimens due to systemic toxicity effects. Because trametinib treatments are associated with cancer cell stemness, its combination with other targeted therapies showing higher cytotoxic effects and, ideally, targeting ovarian CSCs may be key in treatment of HGSOC. Because cells surviving trametinib treatment display very high ALDH1A expression and activity, combination with ALDH inhibitors may also offer benefit. This suggestion is further supported by increases in nifuroxazide sensitivity in melanoma cells overexpressing ALDH1 due to MEK1/2 inhibition [73]. Recently a selective inhibitor of the ALDH1A family was identified as a chemical agent capable of efficiently inducing necroptotic death in ovarian CSCs [22]. The combination of trametinib and an ALDH1A inhibitor could retain the tumor growth arresting effect of trametinib while further complementing it by eliminating the surviving ALDH+ tumor cells in a targeted manner.

## Data Availability

Data from Ovarian Serous Cystadenocarcinoma (TCGA, Nature 2011) dataset used in present study are available from TCGA Research Network (https://www.cancer.gov/tcga (accessed on 18 October 2018)) through cBioPortal website (https://www.cbioportal.org (accessed on 18 October 2018)) [40,41].

## Supplementary Materials

The following are available online at https://www.mdpi.com/2072-6694/13/6/1369/s1, Figure S1. Immunohistochemical staining of phosphorylated ERK1/2 in all available clinical samples of HGSOC and normal fallopian tube tissues, Figure S2. Hematoxylin and eosin staining of all available clinical samples of HGSOC and normal fallopian tube tissues, Figure S3. High-resolution images of phosphorylated ERK1/2 and hematoxylin and eosin staining for patient A19 clinical samples of HGSOC and normal fallopian tube tissues, Figure S4. Response of HGSOC cell lines OVCAR8 and PEO4 to transient or prolonged cisplatin treatment, Figure S5. MEK1/2-ERK1/2 pathway activity changes in cisplatin-resistant HGSOC cell cultures, Figure S6. Changes in functional characteristics of HGSOC cell cultures caused by trametinib treatment, Figure S7. Effect of cell death-related treatment of HGSOC cells, Figure S8. Effects of trametinib washout upon properties of PEO4 cells, Figure S9. Effects of trametinib washout upon properties of OVCAR8 cells, Figure S10. Stemness-related properties of OVCAR8-Washout cells grown in non-adherent 3D conditions, Figure S11. Hematoxylin and eosin staining of PEO4 xenograft tissue samples, Figure S12. Effects of trametinib treatment on MEK1/2 pathway activity in HGSOC in vivo, Figure S13. Effects of trametinib treatment on ALDH1 expression in HGSOC xenografts, uncropped immunoblotting images, Table S1: List of primers used in RT-qPCR analysis, Table S2: Main elements of MEK1/2-ERK1/2 signaling pathway, Table S3: MEK-responsive gene signature.

## References

[1] Siegel R.L., Miller K.D., Jemal A. (2020). Cancer statistics, 2020. CA Cancer J. Clin..

[2] Torre L.A., Trabert B., DeSantis C.E., Miller K.D., Samimi G., Runowicz C.D., Gaudet M.M., Jemal A., Siegel R.L. (2018). Ovarian cancer statistics, 2018. CA Cancer J. Clin..

[3] Kurman R.J., Shih I.M. (2016). The dualistic model of ovarian carcinogenesis: Revisited, revised, and expanded. Am. J. Pathol..

[4] Cooke S.L., Brenton J.D. (2011). Evolution of platinum resistance in high-grade serous ovarian cancer. Lancet Oncol..

[5] Vaughan S., Coward J.I., Bast R.C., Berchuck A., Berek J.S., Brenton J.D., Coukos G., Crum C.C., Drapkin R., Etemadmoghadam D. (2011). Rethinking ovarian cancer: Recommendations for improving outcomes. Nat. Rev. Cancer.

[6] Morgan R.J., Armstrong D.K., Alvarez R.D., Bakkum-Gamez J.N., Behbakht K., Chen L.M., Copeland L., Crispens M.A., DeRosa M., Dorigo O. (2016). Ovarian cancer, version 1.2016, nccn clinical practice guidelines in oncology. J. Natl. Compr. Canc. Netw..

[7] Raja F.A., Chopra N., Ledermann J.A. (2012). Optimal first-line treatment in ovarian cancer. Ann. Oncol..

[8] Davis A., Tinker A.V., Friedlander M. (2014). “Platinum resistant” ovarian cancer: What is it, who to treat and how to measure benefit?. Gynecol. Oncol..

[9] Cortez A.J., Tudrej P., Kujawa K.A., Lisowska K.M. (2018). Advances in ovarian cancer therapy. Cancer Chemother. Pharmacol..

[10] Christie E.L., Bowtell D.D.L. (2017). Acquired chemotherapy resistance in ovarian cancer. Ann. Oncol..

[11] Agarwal R., Kaye S.B. (2003). Ovarian cancer: Strategies for overcoming resistance to chemotherapy. Nat. Rev. Cancer.

[12] Freimund A.E., Beach J.A., Christie E.L., Bowtell D.D.L. (2018). Mechanisms of drug resistance in high-grade serous ovarian cancer. Hematol. Oncol. Clin. N. Am..

[13] Norouzi-Barough L., Sarookhani M.R., Sharifi M., Moghbelinejad S., Jangjoo S., Salehi R. (2018). Molecular mechanisms of drug resistance in ovarian cancer. J. Cell. Physiol..

[14] Mittempergher L. (2016). Genomic characterization of high-grade serous ovarian cancer: Dissecting its molecular heterogeneity as a road towards effective therapeutic strategies. Curr. Oncol. Rep..

[15] Yin X., Jing Y., Cai M.C., Ma P., Zhang Y., Xu C., Zhang M., Di W., Zhuang G. (2017). Clonality, heterogeneity, and evolution of synchronous bilateral ovarian cancer. Cancer Res..

[16] Cooke S.L., Ng C.K., Melnyk N., Garcia M.J., Hardcastle T., Temple J., Langdon S., Huntsman D., Brenton J.D. (2010). Genomic analysis of genetic heterogeneity and evolution in high-grade serous ovarian carcinoma. Oncogene.

[17] Chefetz I., Alvero A.B., Holmberg J.C., Lebowitz N., Craveiro V., Yang-Hartwich Y., Yin G., Squillace L., Gurrea Soteras M., Aldo P. (2013). Tlr2 enhances ovarian cancer stem cell self-renewal and promotes tumor repair and recurrence. Cell Cycle.

[18] Mor G., Yin G., Chefetz I., Yang Y., Alvero A. (2011). Ovarian cancer stem cells and inflammation. Cancer Biol. Ther..

[19] Zhang Q.H., Dou H.T., Xu P., Zhuang S.C., Liu P.S. (2015). Tumor recurrence and drug resistance properties of side population cells in high grade ovary cancer. Drug Res. (Stuttg.).

[20] Kuroda T., Hirohashi Y., Torigoe T., Yasuda K., Takahashi A., Asanuma H., Morita R., Mariya T., Asano T., Mizuuchi M. (2013). Aldh1-high ovarian cancer stem-like cells can be isolated from serous and clear cell adenocarcinoma cells, and aldh1 high expression is associated with poor prognosis. PLoS ONE.

[21] Ruscito I., Cacsire Castillo-Tong D., Vergote I., Ignat I., Stanske M., Vanderstichele A., Ganapathi R.N., Glajzer J., Kulbe H., Trillsch F. (2017). Exploring the clonal evolution of cd133/aldehyde-dehydrogenase-1 (aldh1)-positive cancer stem-like cells from primary to recurrent high-grade serous ovarian cancer (hgsoc). A study of the ovarian cancer therapy-innovative models prolong survival (octips) consortium. Eur. J. Cancer.

[22] Chefetz I., Grimley E., Yang K., Hong L., Vinogradova E.V., Suciu R., Kovalenko I., Karnak D., Morgan C.A., Chtcherbinine M. (2019). A pan-aldh1a inhibitor induces necroptosis in ovarian cancer stem-like cells. Cell Rep..

[23] Chefetz I., Holmberg J.C., Alvero A.B., Visintin I., Mor G. (2011). Inhibition of aurora-a kinase induces cell cycle arrest in epithelial ovarian cancer stem cells by affecting nfkb pathway. Cell Cycle.

[24] Takahashi A., Hong L., Chefetz I. (2020). How to win the ovarian cancer stem cell battle: Destroying the roots. Cancer Drug Resist..

[25] O’Connor M.L., Xiang D., Shigdar S., Macdonald J., Li Y., Wang T., Pu C., Wang Z., Qiao L., Duan W. (2014). Cancer stem cells: A contentious hypothesis now moving forward. Cancer Lett..

[26] Dean M., Fojo T., Bates S. (2005). Tumour stem cells and drug resistance. Nat. Rev. Cancer.

[27] Martins-Neves S.R., Cleton-Jansen A.M., Gomes C.M.F. (2018). Therapy-induced enrichment of cancer stem-like cells in solid human tumors: Where do we stand?. Pharmacol. Res..

[28] Rybak A.P., Ingram A.J., Tang D. (2013). Propagation of human prostate cancer stem-like cells occurs through egfr-mediated erk activation. PLoS ONE.

[29] Ahn H.J., Kim G., Park K.S. (2013). Ell3 stimulates proliferation, drug resistance, and cancer stem cell properties of breast cancer cells via a mek/erk-dependent signaling pathway. Biochem. Biophys. Res. Commun..

[30] Wang Y., Lin X., Fu X., Yan W., Lin F., Kuang P., Luo Y., Lin E., Hong X., Wu G. (2018). Long non-coding rna bancr regulates cancer stem cell markers in papillary thyroid cancer via the raf/mek/erk signaling pathway. Oncol. Rep..

[31] Zhang W., Liu H.T. (2002). Mapk signal pathways in the regulation of cell proliferation in mammalian cells. Cell Res..

[32] Burotto M., Chiou V.L., Lee J.M., Kohn E.C. (2014). The mapk pathway across different malignancies: A new perspective. Cancer.

[33] McCubrey J.A., Steelman L.S., Chappell W.H., Abrams S.L., Wong E.W., Chang F., Lehmann B., Terrian D.M., Milella M., Tafuri A. (2007). Roles of the raf/mek/erk pathway in cell growth, malignant transformation and drug resistance. Biochim. Biophys. Acta.

[34] Caunt C.J., Sale M.J., Smith P.D., Cook S.J. (2015). Mek1 and mek2 inhibitors and cancer therapy: The long and winding road. Nat. Rev. Cancer.

[35] Mandal R., Becker S., Strebhardt K. (2016). Stamping out raf and mek1/2 to inhibit the erk1/2 pathway: An emerging threat to anticancer therapy. Oncogene.

[36] Singer G., Oldt R., Cohen Y., Wang B.G., Sidransky D., Kurman R.J., Shih Ie M. (2003). Mutations in braf and kras characterize the development of low-grade ovarian serous carcinoma. J. Natl. Cancer Inst..

[37] Miller C.R., Oliver K.E., Farley J.H. (2014). Mek1/2 inhibitors in the treatment of gynecologic malignancies. Gynecol. Oncol..

[38] Hew K.E., Miller P.C., El-Ashry D., Sun J., Besser A.H., Ince T.A., Gu M., Wei Z., Zhang G., Brafford P. (2016). Mapk activation predicts poor outcome and the mek inhibitor, selumetinib, reverses antiestrogen resistance in er-positive high-grade serous ovarian cancer. Clin. Cancer Res..

[39] Wang J., Zhou J.Y., Wu G.S. (2007). Erk-dependent mkp-1-mediated cisplatin resistance in human ovarian cancer cells. Cancer Res..

[40] Wang J., Wu G.S. (2014). Role of autophagy in cisplatin resistance in ovarian cancer cells. J. Biol. Chem..

[41] Cerami E., Gao J., Dogrusoz U., Gross B.E., Sumer S.O., Aksoy B.A., Jacobsen A., Byrne C.J., Heuer M.L., Larsson E. (2012). The cbio cancer genomics portal: An open platform for exploring multidimensional cancer genomics data. Cancer Discov..

[42] Gao J., Aksoy B.A., Dogrusoz U., Dresdner G., Gross B., Sumer S.O., Sun Y., Jacobsen A., Sinha R., Larsson E. (2013). Integrative analysis of complex cancer genomics and clinical profiles using the cbioportal. Sci. Signal..

[43] Silva I.A., Bai S., McLean K., Yang K., Griffith K., Thomas D., Ginestier C., Johnston C., Kueck A., Reynolds R.K. (2011). Aldehyde dehydrogenase in combination with cd133 defines angiogenic ovarian cancer stem cells that portend poor patient survival. Cancer Res..

[44] Pfaffl M.W. (2001). A new mathematical model for relative quantification in real-time rt-pcr. Nucleic Acids Res..

[45] Rueden C.T., Schindelin J., Hiner M.C., DeZonia B.E., Walter A.E., Arena E.T., Eliceiri K.W. (2017). Imagej2: Imagej for the next generation of scientific image data. BMC Bioinf..

[46] Bengtsson M., Stahlberg A., Rorsman P., Kubista M. (2005). Gene expression profiling in single cells from the pancreatic islets of langerhans reveals lognormal distribution of mrna levels. Genome Res..

[47] Lee S., Yoon S., Kim D.H. (2007). A high nuclear basal level of erk2 phosphorylation contributes to the resistance of cisplatin-resistant human ovarian cancer cells. Gynecol. Oncol..

[48] Kielbik M., Krzyzanowski D., Pawlik B., Klink M. (2018). Cisplatin-induced erk1/2 activity promotes g1 to s phase progression which leads to chemoresistance of ovarian cancer cells. Oncotarget.

[49] Taniguchi T., Tischkowitz M., Ameziane N., Hodgson S.V., Mathew C.G., Joenje H., Mok S.C., D’Andrea A.D. (2003). Disruption of the fanconi anemia-brca pathway in cisplatin-sensitive ovarian tumors. Nat. Med..

[50] Haley J., Tomar S., Pulliam N., Xiong S., Perkins S.M., Karpf A.R., Mitra S., Nephew K.P., Mitra A.K. (2016). Functional characterization of a panel of high-grade serous ovarian cancer cell lines as representative experimental models of the disease. Oncotarget.

[51] Chang K.E., Wei B.R., Madigan J.P., Hall M.D., Simpson R.M., Zhuang Z., Gottesman M.M. (2015). The protein phosphatase 2a inhibitor lb100 sensitizes ovarian carcinoma cells to cisplatin-mediated cytotoxicity. Mol. Cancer Ther..

[52] Wagle M.C., Kirouac D., Klijn C., Liu B., Mahajan S., Junttila M., Moffat J., Merchant M., Huw L., Wongchenko M. (2018). A transcriptional mapk pathway activity score (mpas) is a clinically relevant biomarker in multiple cancer types. NPJ Precis. Oncol..

[53] Khan I., Yousif A., Chesnokov M., Hong L., Chefetz I. (2020). A decade of cell death studies: Breathing new life into necroptosis. Pharmacol. Ther..

[54] Foster R., Buckanovich R.J., Rueda B.R. (2013). Ovarian cancer stem cells: Working towards the root of stemness. Cancer Lett..

[55] Roy L., Cowden Dahl K.D. (2018). Can stemness and chemoresistance be therapeutically targeted via signaling pathways in ovarian cancer?. Cancers.

[56] Dhillon A.S., Hagan S., Rath O., Kolch W. (2007). Map kinase signalling pathways in cancer. Oncogene.

[57] Liu S., Zha J., Lei M. (2018). Inhibiting erk/mnk/eif4e broadly sensitizes ovarian cancer response to chemotherapy. Clin. Transl. Oncol..

[58] Gilmartin A.G., Bleam M.R., Groy A., Moss K.G., Minthorn E.A., Kulkarni S.G., Rominger C.M., Erskine S., Fisher K.E., Yang J. (2011). Gsk1120212 (jtp-74057) is an inhibitor of mek activity and activation with favorable pharmacokinetic properties for sustained in vivo pathway inhibition. Clin. Cancer Res..

[59] Yeh T.C., Marsh V., Bernat B.A., Ballard J., Colwell H., Evans R.J., Parry J., Smith D., Brandhuber B.J., Gross S. (2007). Biological characterization of arry-142886 (azd6244), a potent, highly selective mitogen-activated protein kinase kinase 1/2 inhibitor. Clin. Cancer Res..

[60] Hoeflich K.P., Merchant M., Orr C., Chan J., Den Otter D., Berry L., Kasman I., Koeppen H., Rice K., Yang N.Y. (2012). Intermittent administration of mek inhibitor gdc-0973 plus pi3k inhibitor gdc-0941 triggers robust apoptosis and tumor growth inhibition. Cancer Res..

[61] Simpkins F., Jang K., Yoon H., Hew K.E., Kim M., Azzam D.J., Sun J., Zhao D., Ince T.A., Liu W. (2018). Dual src and mek inhibition decreases ovarian cancer growth and targets tumor initiating stem-like cells. Clin. Cancer Res..

[62] Baba T., Convery P.A., Matsumura N., Whitaker R.S., Kondoh E., Perry T., Huang Z., Bentley R.C., Mori S., Fujii S. (2009). Epigenetic regulation of cd133 and tumorigenicity of cd133+ ovarian cancer cells. Oncogene.

[63] Curley M.D., Therrien V.A., Cummings C.L., Sergent P.A., Koulouris C.R., Friel A.M., Roberts D.J., Seiden M.V., Scadden D.T., Rueda B.R. (2009). Cd133 expression defines a tumor initiating cell population in primary human ovarian cancer. Stem Cells.

[64] Kryczek I., Liu S., Roh M., Vatan L., Szeliga W., Wei S., Banerjee M., Mao Y., Kotarski J., Wicha M.S. (2012). Expression of aldehyde dehydrogenase and cd133 defines ovarian cancer stem cells. Int. J. Cancer.

[65] Choi Y.J., Ingram P.N., Yang K., Coffman L., Iyengar M., Bai S., Thomas D.G., Yoon E., Buckanovich R.J. (2015). Identifying an ovarian cancer cell hierarchy regulated by bone morphogenetic protein 2. Proc. Natl. Acad. Sci. USA.

[66] Fremin C., Meloche S. (2010). From basic research to clinical development of mek1/2 inhibitors for cancer therapy. J. Hematol. Oncol..

[67] Meloche S., Pouyssegur J. (2007). The erk1/2 mitogen-activated protein kinase pathway as a master regulator of the g1- to s-phase transition. Oncogene.

[68] Zhao Y., Adjei A.A. (2014). The clinical development of mek inhibitors. Nat. Rev. Clin. Oncol..

[69] Cappuccio S., Distefano M.G., Ghizzoni V., Fagotti A., Scambia G. (2020). Trametinib response in heavily pretreated high-grade ovarian cancer: One step towards precision medicine. Gynecol. Oncol. Rep..

[70] Thigpen J.T., Blessing J.A., Ball H., Hummel S.J., Barrett R.J. (1994). Phase ii trial of paclitaxel in patients with progressive ovarian carcinoma after platinum-based chemotherapy: A gynecologic oncology group study. J. Clin. Oncol..

[71] Jordan M.A., Toso R.J., Thrower D., Wilson L. (1993). Mechanism of mitotic block and inhibition of cell proliferation by taxol at low concentrations. Proc. Natl. Acad. Sci. USA.

[72] Takada M., Hix J.M.L., Corner S., Schall P.Z., Kiupel M., Yuzbasiyan-Gurkan V. (2018). Targeting mek in a translational model of histiocytic sarcoma. Mol. Cancer Ther..

[73] Sumi T., Hirai S., Yamaguchi M., Tanaka Y., Tada M., Niki T., Takahashi H., Sakuma Y. (2018). Trametinib downregulates survivin expression in rb1-positive kras-mutant lung adenocarcinoma cells. Biochem. Biophys. Res. Commun..

[74] Vogel C.J., Smit M.A., Maddalo G., Possik P.A., Sparidans R.W., van der Burg S.H., Verdegaal E.M., Heck A.J., Samatar A.A., Beijnen J.H. (2015). Cooperative induction of apoptosis in nras mutant melanoma by inhibition of mek and rock. Pigment Cell Melanoma Res..

[75] Fernandez M.L., DiMattia G.E., Dawson A., Bamford S., Anderson S., Hennessy B.T., Anglesio M.S., Shepherd T.G., Salamanca C., Hoenisch J. (2016). Differences in mek inhibitor efficacy in molecularly characterized low-grade serous ovarian cancer cell lines. Am. J. Cancer Res..

[76] Zhan T., Ambrosi G., Wandmacher A.M., Rauscher B., Betge J., Rindtorff N., Häussler R.S., Hinsenkamp I., Bamberg L., Hessling B. (2019). Mek inhibitors activate wnt signalling and induce stem cell plasticity in colorectal cancer. Nat. Commun..

[77] Sarvi S., Crispin R., Lu Y., Zeng L., Hurley T.D., Houston D.R., von Kriegsheim A., Chen C.H., Mochly-Rosen D., Ranzani M. (2018). Aldh1 bio-activates nifuroxazide to eradicate aldh(high) melanoma-initiating cells. Cell Chem. Biol..

[78] Zhang J., Guo X., Chang D.Y., Rosen D.G., Mercado-Uribe I., Liu J. (2012). Cd133 expression associated with poor prognosis in ovarian cancer. Mod. Pathol..

[79] Liang J., Yang B., Cao Q., Wu X. (2016). Association of vasculogenic mimicry formation and cd133 expression with poor prognosis in ovarian cancer. Gynecol. Obstet. Investig..

[80] Stemberger-Papic S., Vrdoljak-Mozetic D., Ostojic D.V., Rubesa-Mihaljevic R., Krigtofic I., Brncic-Fisher A., Kragevic M., Eminovic S. (2015). Expression of cd133 and cd117 in 64 serous ovarian cancer cases. Coll. Antropol..

[81] Steg A.D., Bevis K.S., Katre A.A., Ziebarth A., Dobbin Z.C., Alvarez R.D., Zhang K., Conner M., Landen C.N. (2012). Stem cell pathways contribute to clinical chemoresistance in ovarian cancer. Clin. Cancer Res..

[82] Xia Y., Wei X., Gong H., Ni Y. (2018). Aldehyde dehydrogenase serves as a biomarker for worse survival profiles in ovarian cancer patients: An updated meta-analysis. BMC Womens Health.

[83] Tolcher A.W., Bendell J.C., Papadopoulos K.P., Burris H.A., Patnaik A., Jones S.F., Rasco D., Cox D.S., Durante M., Bellew K.M. (2015). A phase ib trial of the oral mek inhibitor trametinib (gsk1120212) in combination with everolimus in patients with advanced solid tumors. Ann. Oncol..

[84] Ikeda M., Ioka T., Fukutomi A., Morizane C., Kasuga A., Takahashi H., Todaka A., Okusaka T., Creasy C.L., Gorman S. (2018). Efficacy and safety of trametinib in japanese patients with advanced biliary tract cancers refractory to gemcitabine. Cancer Sci..

