# Supplementary material for: The MEK1/2 Pathway as a Therapeutic Target in High-Grade Serous Ovarian Carcinoma
Source: Cancers (Basel). 2021 Mar 18;13(6):1369. doi: 10.3390/cancers13061369 (PMC8003094; doi:10.3390/cancers13061369)
Supplement: Supplementary file 1 [file cancers-13-01369-s001.pdf]

Table S1. List of primers used in RT-qPCR analysis.

Gene	Forward primer	Reverse primer
TBP	GAGAGTTCTGGGATTGTACC	GGATTATATTCGGCGTTTCG
PHLDA1	GGCAAGACAAGGTTTTGAGG	TCGCAAGTTTTCAGTAGGGTG
SPRY2	TCCACTCAGCACAAACAC	GATTATGCCATCAGCAACAG
SPRY4	CAACGGCTCTTAGACCAC	CACACTCCTTGCATTTACAC
DUSP4	CCCCACTACACGACCAG	TCCGAGGAGACATTCAACAG
DUSP6	GACGCTCGCTGTTTGTATC	GCTTCTAATCCCTCCCTCC
CCND1	CTGGATGCTGGAGGTCTG	GGTCTCCTTCATCTTAGAGGC
EPHA2	GTCAGCATCAACCAGACAGA	TCCCTTCTTGCGGTAAGTG
EPHA4	CGGAGCGGAGAATGC	TCCTTCCCAGACAGAGTAG
ETV4	AGGAGACATCAAGCAGGAAGGG	CCCAGAGCCTGGCGACC
ETV5	AGCACAAGTTCCTGATGATG	CATAGTTAGCACCAAGAGCC
SOX2	CACATGAACGGCTGGAG	CTGGTCATGGAGTTGTACTG
NANOG	AACTCTCCAACATCCTGAAC	GTAGGAAGAGTAAAGGCTGG
POU5F1	GGGAAGGTATTCAGCCAAAC	AGAACCACACTCGGACC
KLF4	CACACGGGATGATGCTC	GTCACAGTGGTAAGGTTTCT
ALDH1A1	TGTTAGCTGATGCCGACTTG	TTCTTAGCCCGCTCAACACT
ALDH1A2	CCATTGGAGTGTGTGGACAG	GATGAGGGCTCCCATGTAGA
ALDH1A3	TCTCGACAAAGCCCTGAAGT	GTCCGATGTTTGAGGAAGGA

Table S2. Main elements of MEK1/2-ERK1/2 signaling pathway.

Protein name	UniProt ID	Gene name	NCBI Gene ID	Function	References
GRB2	P62993	*GRB2*	2885	Adaptor protein linking membrane receptors and RAS-controlled signaling pathways	[85-88]
SOS1	Q07889	*SOS1*	6654	Promoter of GDP-GTP exchange in RAS proteins	[89]
KRas<br>HRas<br>NRas	P01116<br>P01112<br>P01111	*KRAS*<br>*HRAS*<br>*NRAS*	3845<br>3265<br>4893	Family of membrane-bound GTPases mediating signal transduction from GRB2 to RAF proteins	[90-93]
BRAF<br>RAF1	P15056<br>P04049	*BRAF*<br>*RAF1*	673<br>5894	Family of protein kinases activated by Ras proteins and targeting MEK1/2	[91,94-98]
MEK1<br>MEK2	Q01986<br>P36507	*MAP2K1*<br>*MAP2K2*	5604<br>5605	Protein kinases, central elements of MAP kinase signaling cascade targeting ERK1/2	[94,99,100]
ERK1<br>ERK2	P27361<br>P28482	*MAPK3*<br>*MAPK1*	5595<br>5594	Protein kinases, main effectors of MAP kinase signaling cascade, phosphorylate more than 100 proteins	[94,99,101]

Table S3. MEK-responsive gene signature [52].

Gene name	NCBI Gene ID	Function	References
PHLDA1	22822	Apoptosis regulator, may exert both pro- and anti-apoptotic effects in different tumors	[102,103]
SPRY2	10253	Proliferation suppressor, negative feedback regulator of MEK-ERK activity, promotes glioblastoma growth and chemoresistance	[104-106]
SPRY4	81848	Proliferation suppressor, negative feedback regulator of MEK-ERK activity	[107,108]
DUSP4	1846	Proliferation and invasion suppressor, ERK phosphorylation inhibitor	[109,110]
DUSP6	1848	Proliferation suppressor, metastases formation suppressor, ERK phosphorylation inhibitor	[111,112]
CCND1	595	Proliferation stimulator, proto-oncogene	[113,114]
EPHA2	1969	Proliferation, EMT and invasion stimulator, apoptosis stimulator in some systems	[115-117]
EPHA4	2043	Invasion stimulator, marker of poor prognosis	[118,119]
ETV4	2118	Proliferation and invasion stimulator, sustains cell stemness	[120-122]
ETV5	2119	Proliferation stimulator, invasion suppressor, sustains cell stemness	[122-124]

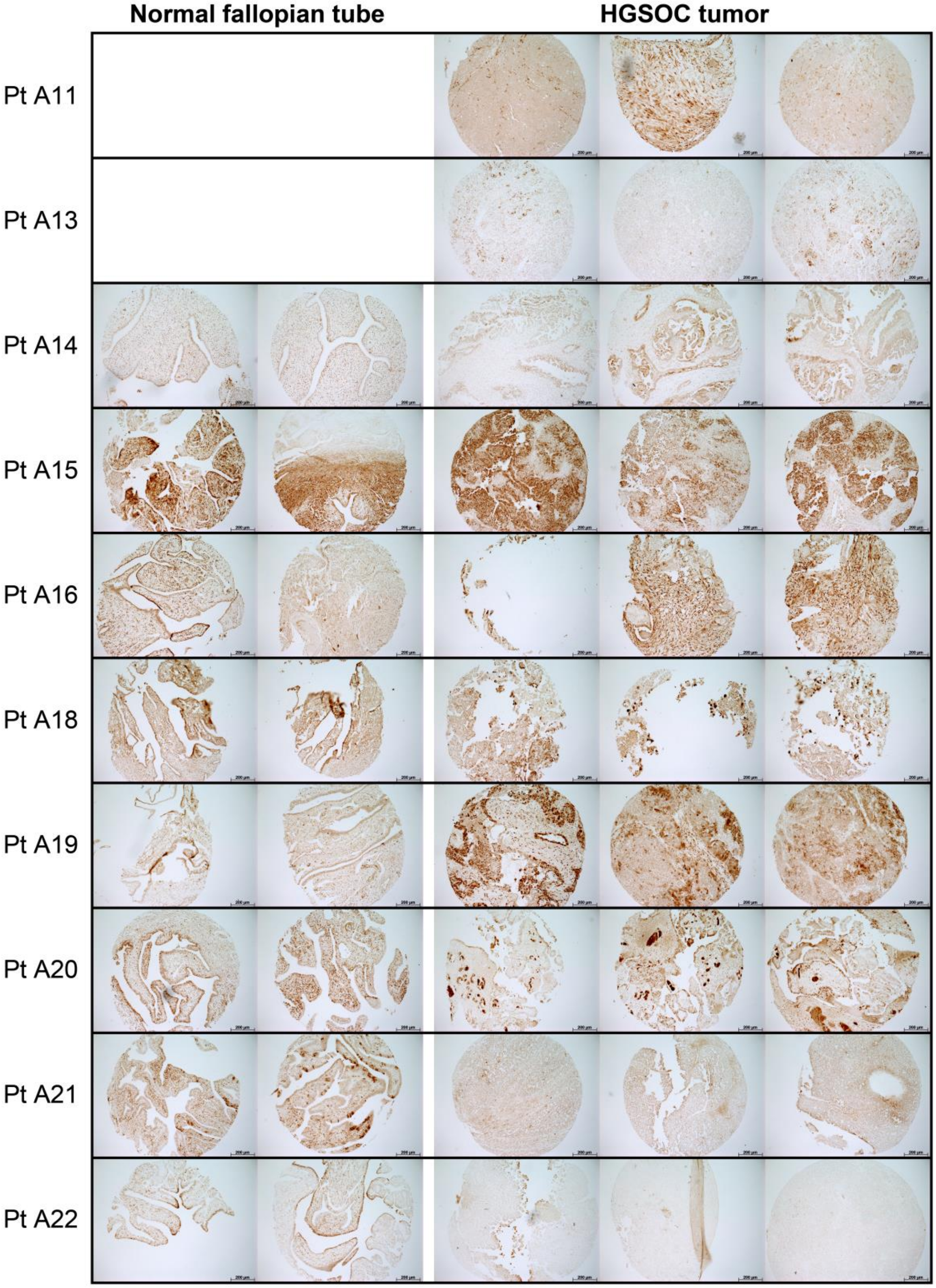

Figure S1. Immunohistochemical staining of phosphorylated ERK1/2 in all available clinical samples of HGSOC and normal fallopian tube tissues. Scale bars: 200 μm. Pt XXX – patient number XXX as provided by Lifespan Research Laboratories.

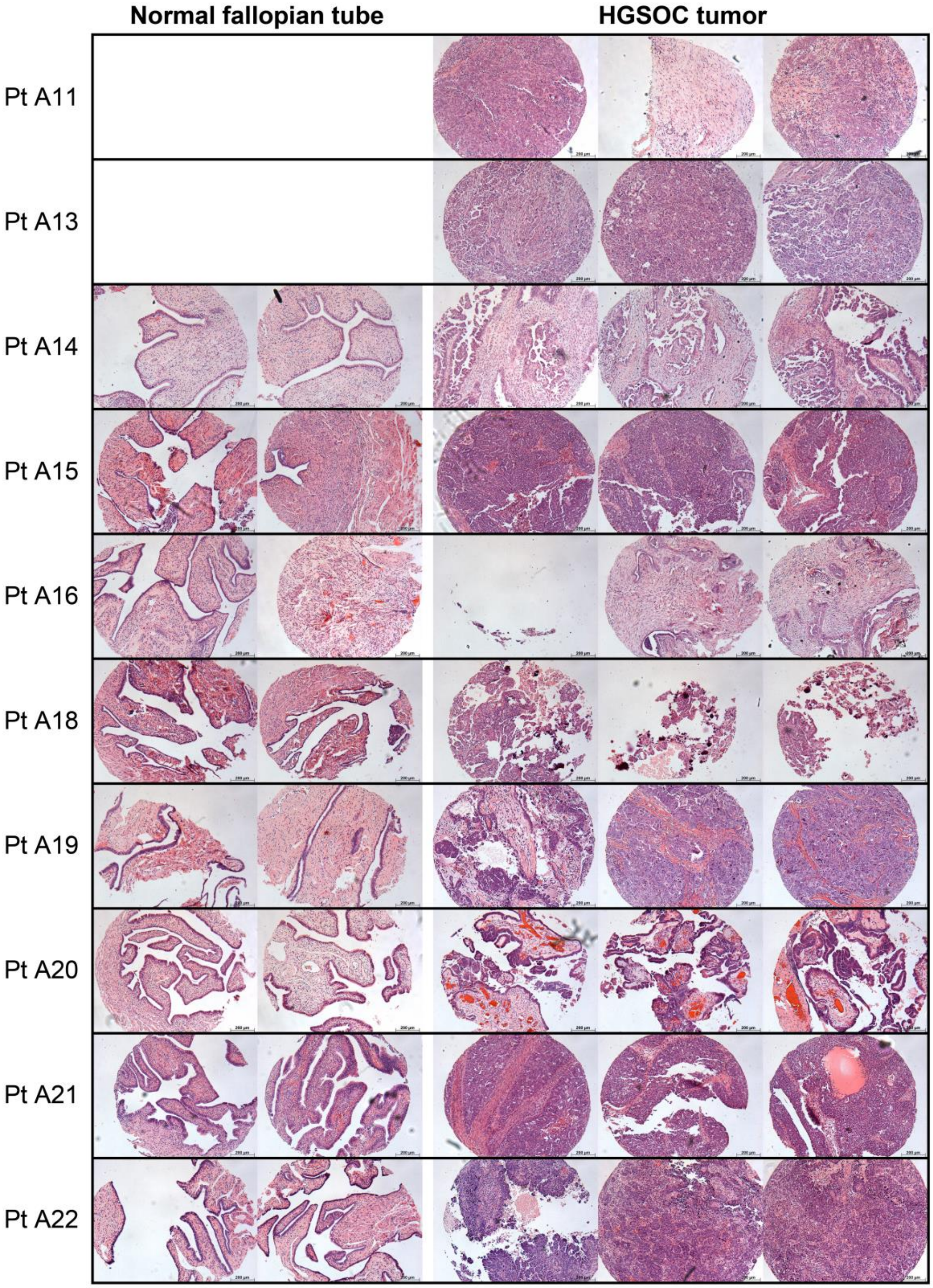

Figure S2. Hematoxylin and eosin staining of all available clinical samples of HGSOC and normal fallopian tube tissues. Scale bars: 200 μm. Pt XXX – patient number XXX as provided by Lifespan Research Laboratories.

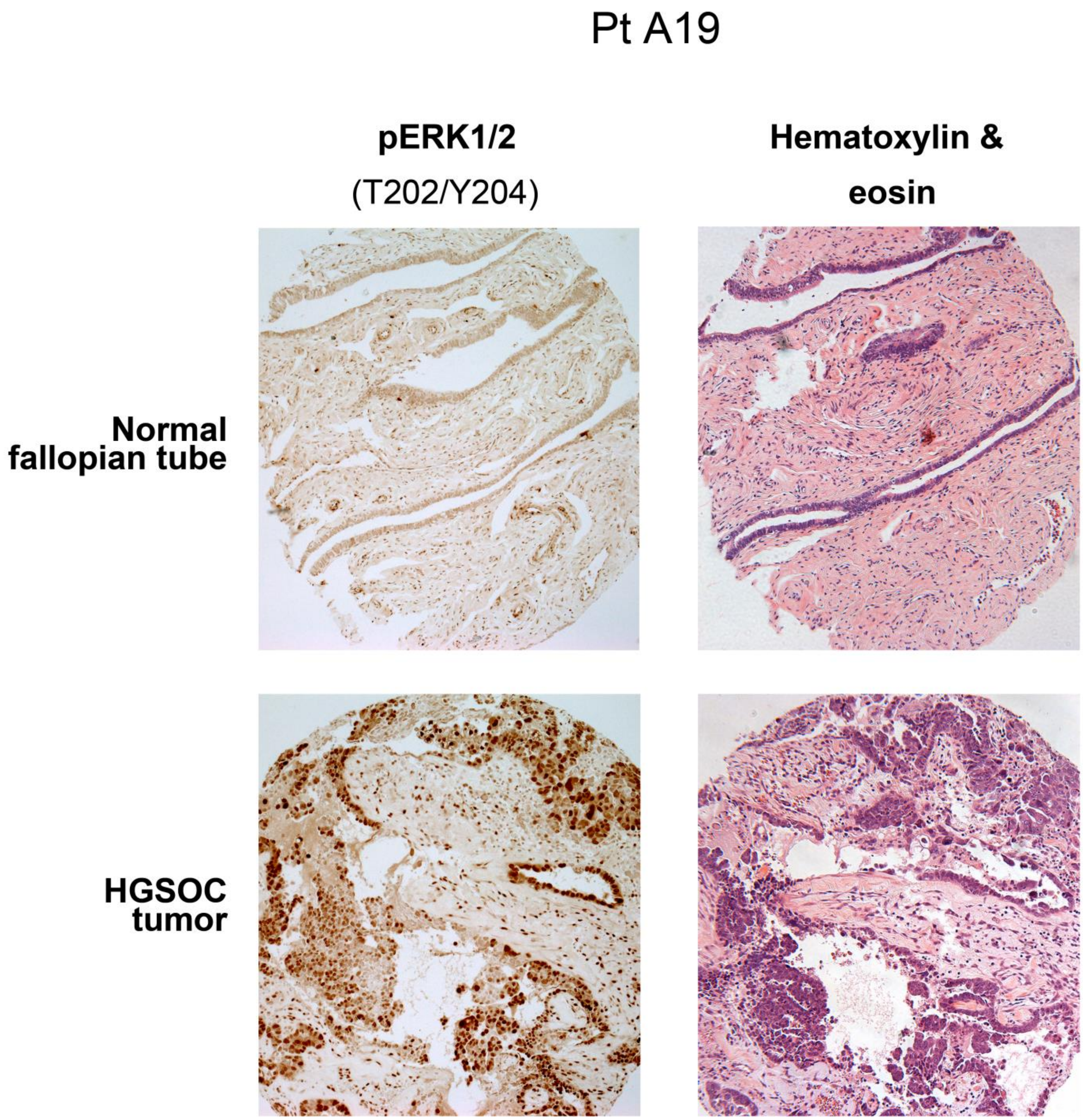

Figure S3. High-resolution images of phosphorylated ERK1/2 and hematoxylin and eosin staining for patient A19 clinical samples of HGSOC and normal fallopian tube tissues. Scale bars: 200 µm. Pt A19 - patient number A19 as provided by Lifespan Research Laboratories, pERK1/2 – phosphorylated ERK1/2.

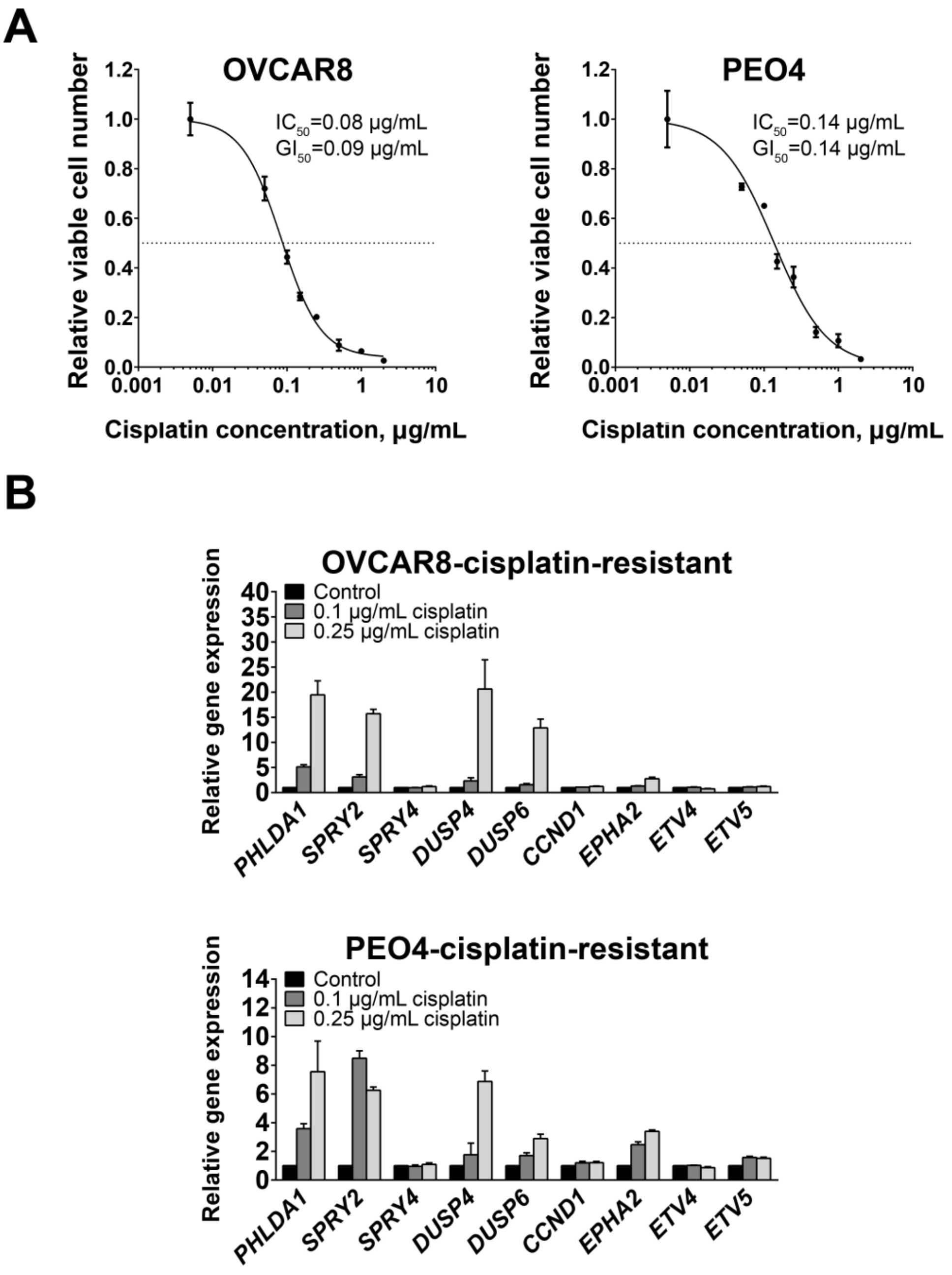

Figure S4. Response of HGSOC cell lines OVCAR8 and PEO4 to transient or prolonged cisplatin treatment. (A) Dose-response curves generated using relative viable cell numbers after treatment with various concentrations of cisplatin for 72 hours. Data are normalized to vehicle-treated control samples (not shown) and presented as mean±S.D. (N=3). **(B)** Gene expression levels of MEK1/2-responsive genes in cells resistant to the indicated concentrations of cisplatin. Data are normalized to "Control" samples and presented as mean+S.E.M. (N=3, technical replicates).

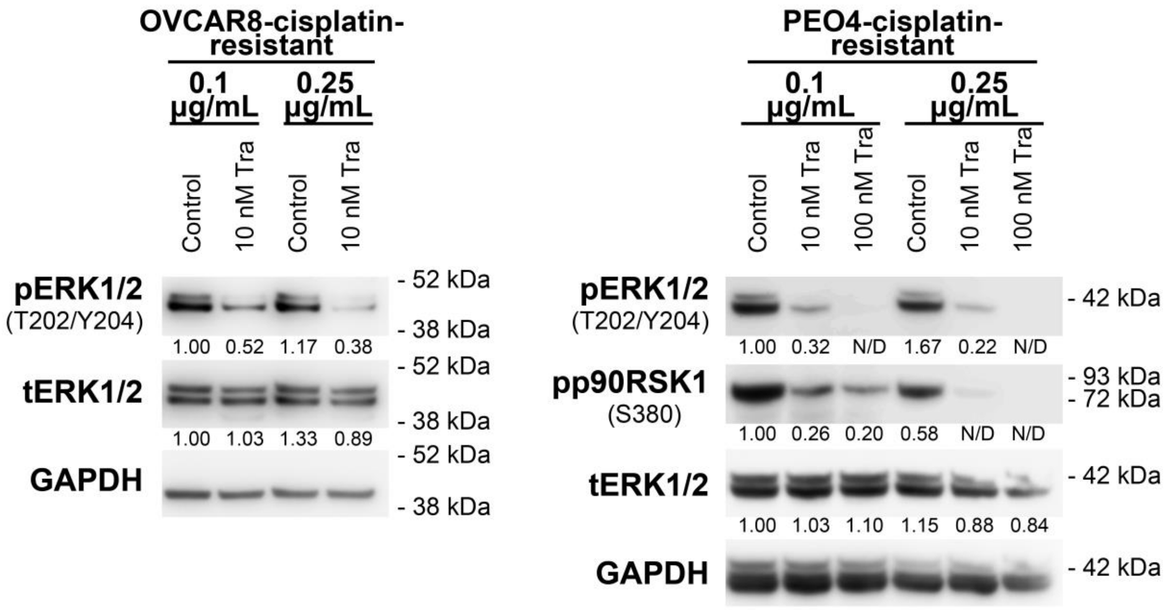

Figure S5. MEK1/2-ERK1/2 pathway activity changes in cisplatin-resistant HGSOC cell cultures. Immunoblotting analysis of phosphorylated and total ERK1/2 protein levels in cisplatin-resistant cells treated with trametinib for 24 hours. Numbers under the bands represent relative intensity normalized to GAPDH levels and "0.1 µg/mL-Control" samples. Tra – trametinib, pERK1/2 – phosphorylated ERK1/2, pp90RSK1 – phosphorylated p90RSK1, tERK1/2 – total ERK1/2, N/D – non-detectable signal.

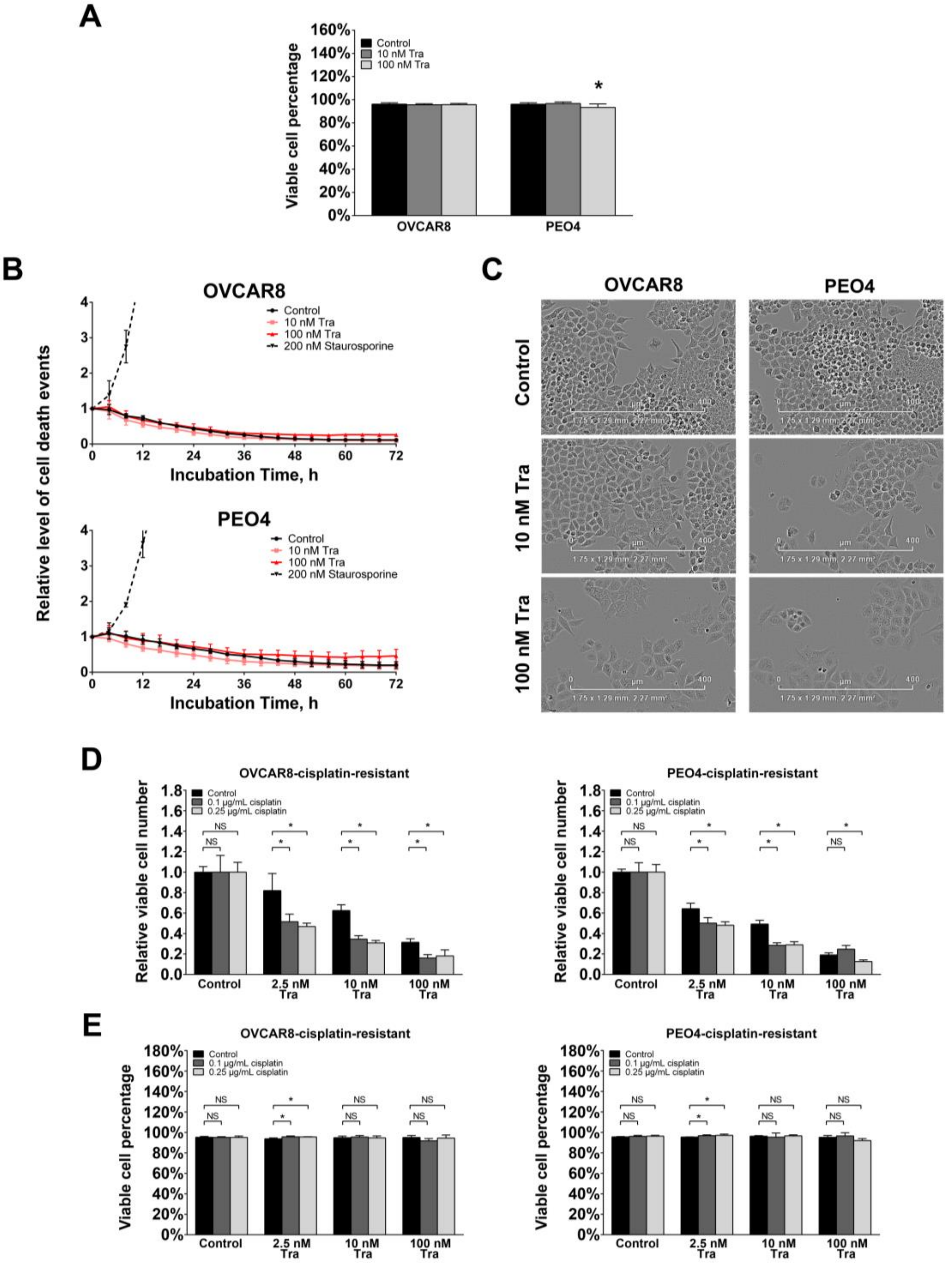

Figure S6. Changes in functional characteristics of HGSOC cell cultures caused by trametinib treatment. (A) Viable cell percentages after treatment with trametinib for 72 hours. Data are presented as mean+S.D. (N=6, Mann-Whitney U-test). **(B)** Cytotoxic effect of trametinib treatment detected using the CytoTox reagent. Staurosporin is used as a positive control. Fluorescence level for each time point is normalized to the area covered by cells and starting value; data are presented as mean±S.D. (N=4, Mann-Whitney U-test). **(C)** Cell morphology and confluence after treatment with trametinib for 72 hours. Scale bars: 400 μm. **(D)** Viable cell numbers of cisplatin-resistant cells after treatment with trametinib for 72 hours. Data are normalized to "Control" samples for each cell culture and presented as mean+S.D. (N=4, Mann-Whitney U-test). **(E)** Viable cell percentages of cisplatin-resistant cells after treatment with trametinib for 72 hours. Data are presented as mean+S.D. (N=4, Mann-Whitney U-test). Tra – trametinib, NS – non-significant difference.

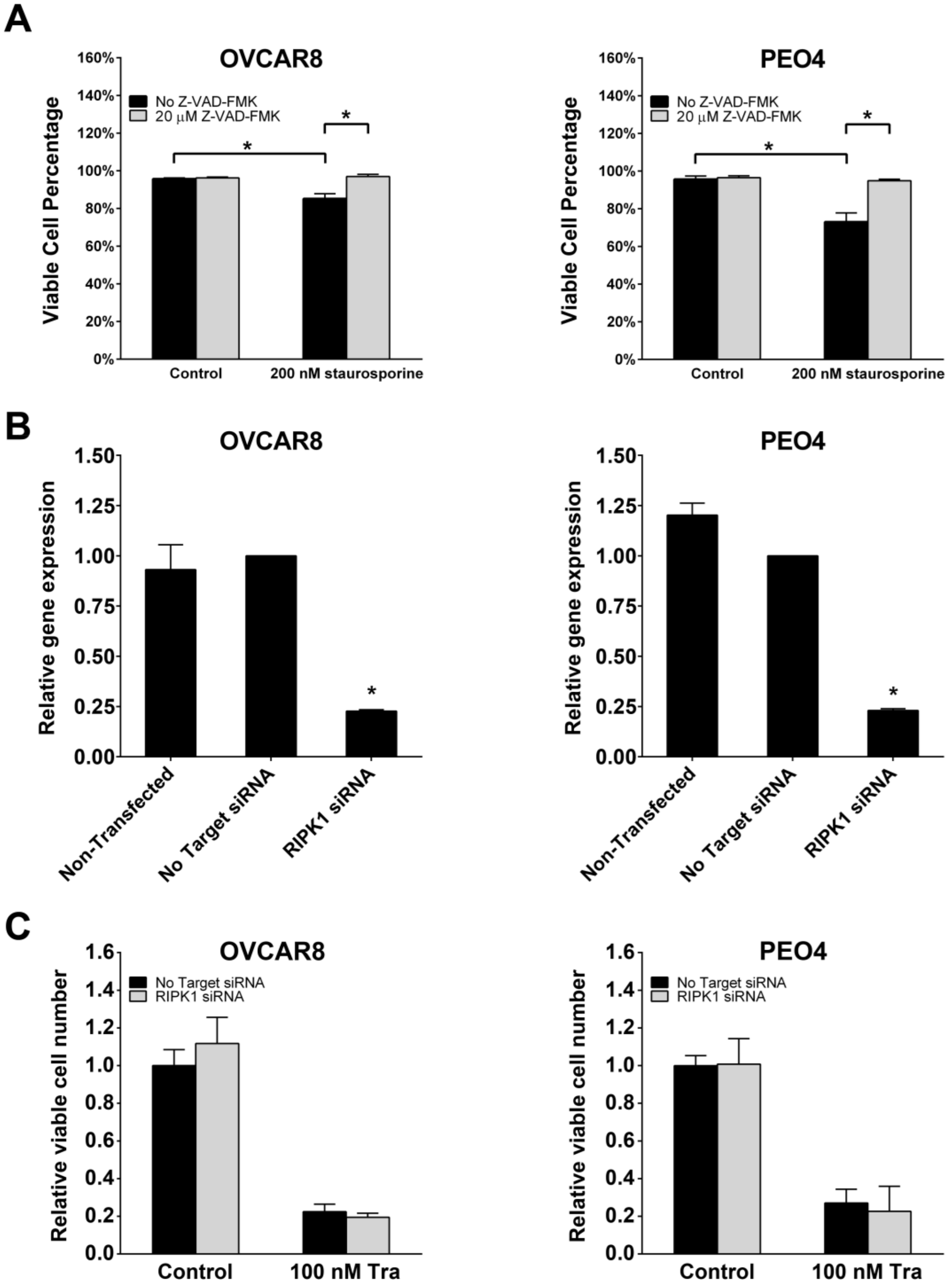

Figure S7. Effect of cell death-related treatment of HGSOC cells. (A) Viable cell percentages of cells treated with pan-caspase inhibitor Z-VAD-FMK and staurosporine for 24 hours. Data are normalized to "Control, No Z-VAD-FMK" sample and presented as mean+S.D. (N=3, one-tailed Mann-Whitney U-test, * – $p = 0.05$). **(B)** Gene expression levels of *RIPK1* gene in cells transfected with anti-*RIPK1* siRNA. Data are normalized to "No Target siRNA" samples and presented as mean+S.D. (N=3, two-tailed Student's T-test with Welch's correction). **(C)** Viable cell numbers of cells with siRNA-mediated *RIPK1* knockdown treated with trametinib for 72 hours. Data are normalized to "Control, No Target siRNA" sample and presented as mean+S.D. (N=3, no statistical analysis performed due to insufficient number of replicate and clear lack of differences). Tra – trametinib, siRNA – short interfering RNA.

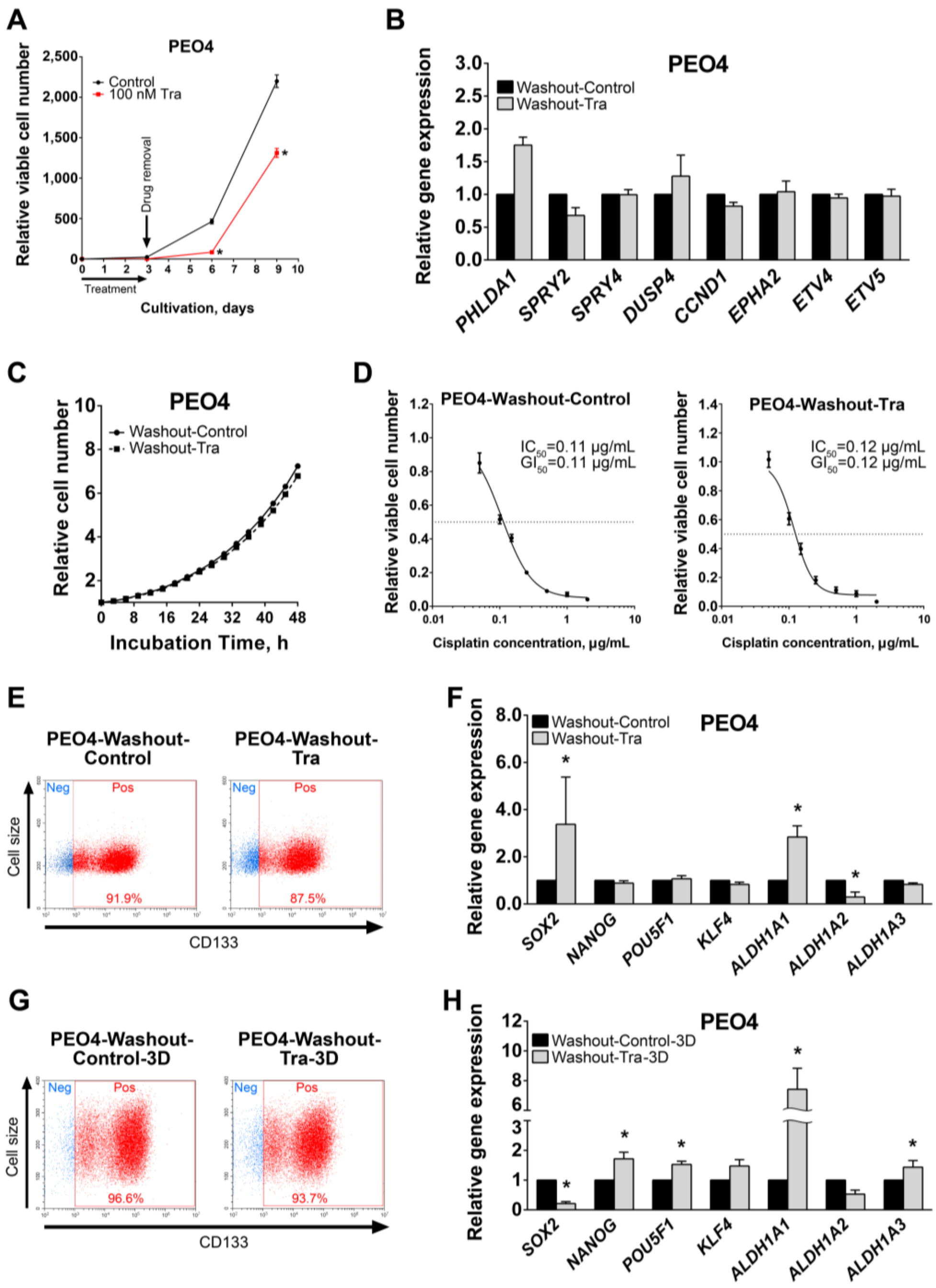

Figure S8. Effects of trametinib washout upon properties of PEO4 cells. (A) Cell growth kinetics during the initial establishment of PEO4-Washout cells. Data are normalized to starting cell number and presented as mean±S.D. (N=6, Mann-Whitney U-test). **(B)** Gene expression levels of MEK1/2-responsive genes. Data are normalized to "Washout-Control" samples and presented as mean+S.E.M. (N=3, technical replicates). **(C)** Growth kinetics of cells in standard conditions. Data are normalized to starting cell number and presented as mean±S.D. (N=4, Mann-Whitney U-test). **(D)** Dose-response curves generated using relative viable numbers of cells after treatment with various concentrations of cisplatin for 72 hours. Data are normalized to vehicle-treated control samples (not shown) and presented as mean±S.D. (N=3). **(E)** Expression of the CD133 surface marker in cells grown in standard conditions. Gates indicate CD133-negative ("Neg") and CD133-positive ("Pos") cell subpopulations. **(F)** Gene expression levels of stemness-related genes. Data are normalized to "Washout-Control" samples and presented as mean+S.D. (N=3, two-tailed Student's T-test with Welch's correction). **(G)** Expression of the CD133 surface marker in cells grown in non-adherent conditions. Gates indicate CD133-negative ("Neg") and CD133-positive ("Pos") cell subpopulations. **(H)** Gene expression levels of stemness-related genes in cells grown in non-adherent conditions. Data are normalized to "Washout-Control" samples and presented as mean+S.D. (N=3, two-tailed Student's T-test with Welch's correction). Tra – trametinib.

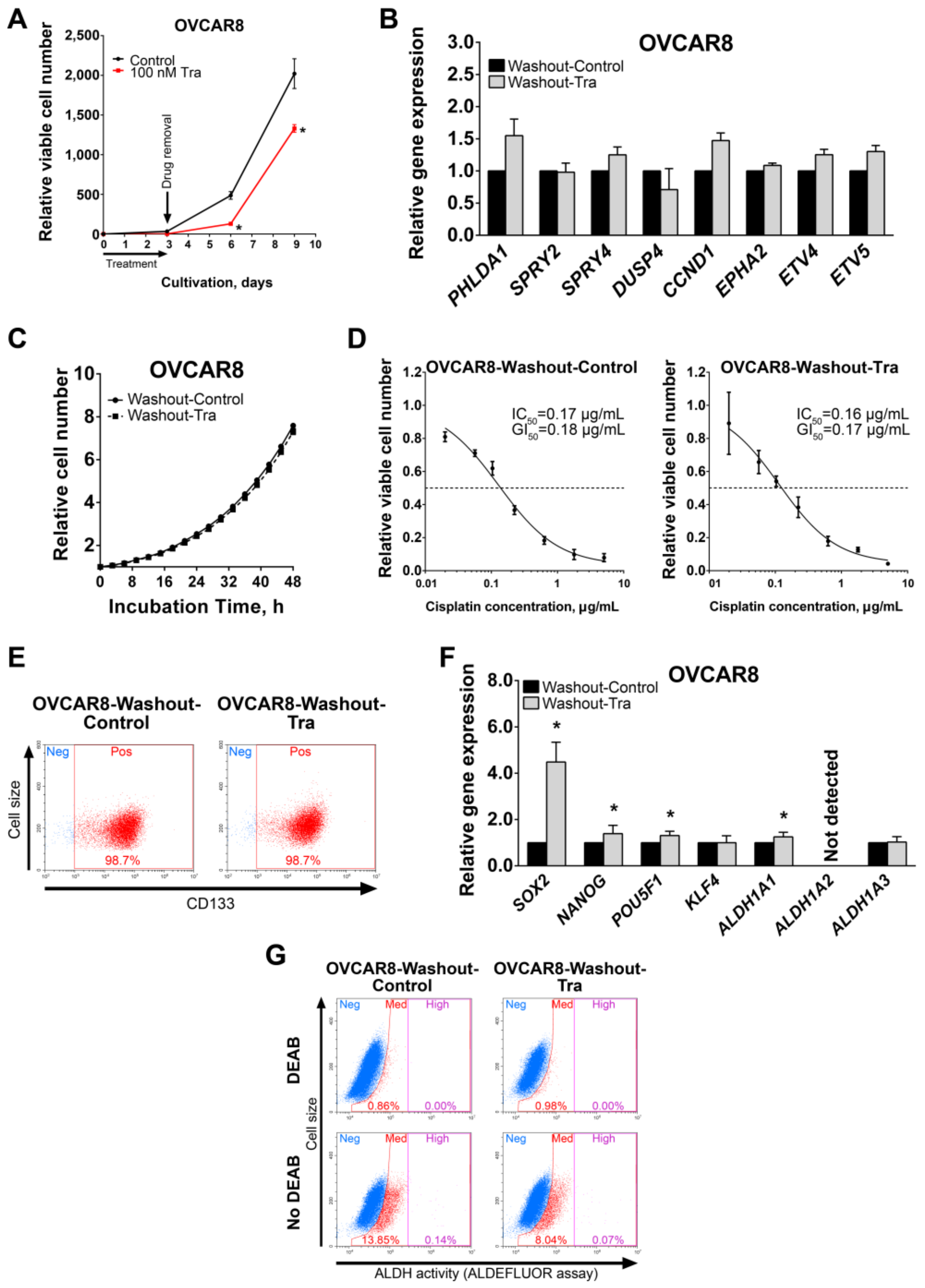

Figure S9. Effects of trametinib washout upon properties of OVCAR8 cells. (A) Cell growth kinetics during the initial establishment of OVCAR8-Washout cells. Data are normalized to starting cell number and presented as mean±S.D. (N=6, Mann-Whitney U-test). **(B)** Gene expression levels of MEK1/2-responsive genes. Data are normalized to "Washout-Control" samples and presented as mean+S.E.M. (N=3, technical replicates). **(C)** Growth kinetics of cells in standard conditions. Data are normalized to starting cell number and presented as mean±S.D. (N=4, Mann-Whitney U-test). **(D)** Dose-response curves generated using relative viable numbers of cells after treatment with various concentrations of cisplatin for 72 hours. Data are normalized to vehicle-treated control samples (not shown) and presented as mean±S.D. (N=3). **(E)** Expression of the CD133 surface marker in cells grown in standard conditions. Gates indicate CD133-negative ("Neg") and CD133-positive ("Pos") cell subpopulations. **(F)** Gene expression levels of stemness-related genes. Data are normalized to "Washout-Control" samples and presented as mean+S.D. (N=3, two-tailed Student's T-test with Welch's correction). **(G)** ALDEFLUOR analysis of cells after cultivation in non-adherent 3D conditions for 7 days. DEAB was used as ALDH inhibitor to define background ALDEFLUOR signal and set proper gates for ALDH-positive cells. Gates indicate cell subpopulations displaying negative ("Neg"), medium ("Med"), or high ("High") levels of ALDH activity. Tra – trametinib.

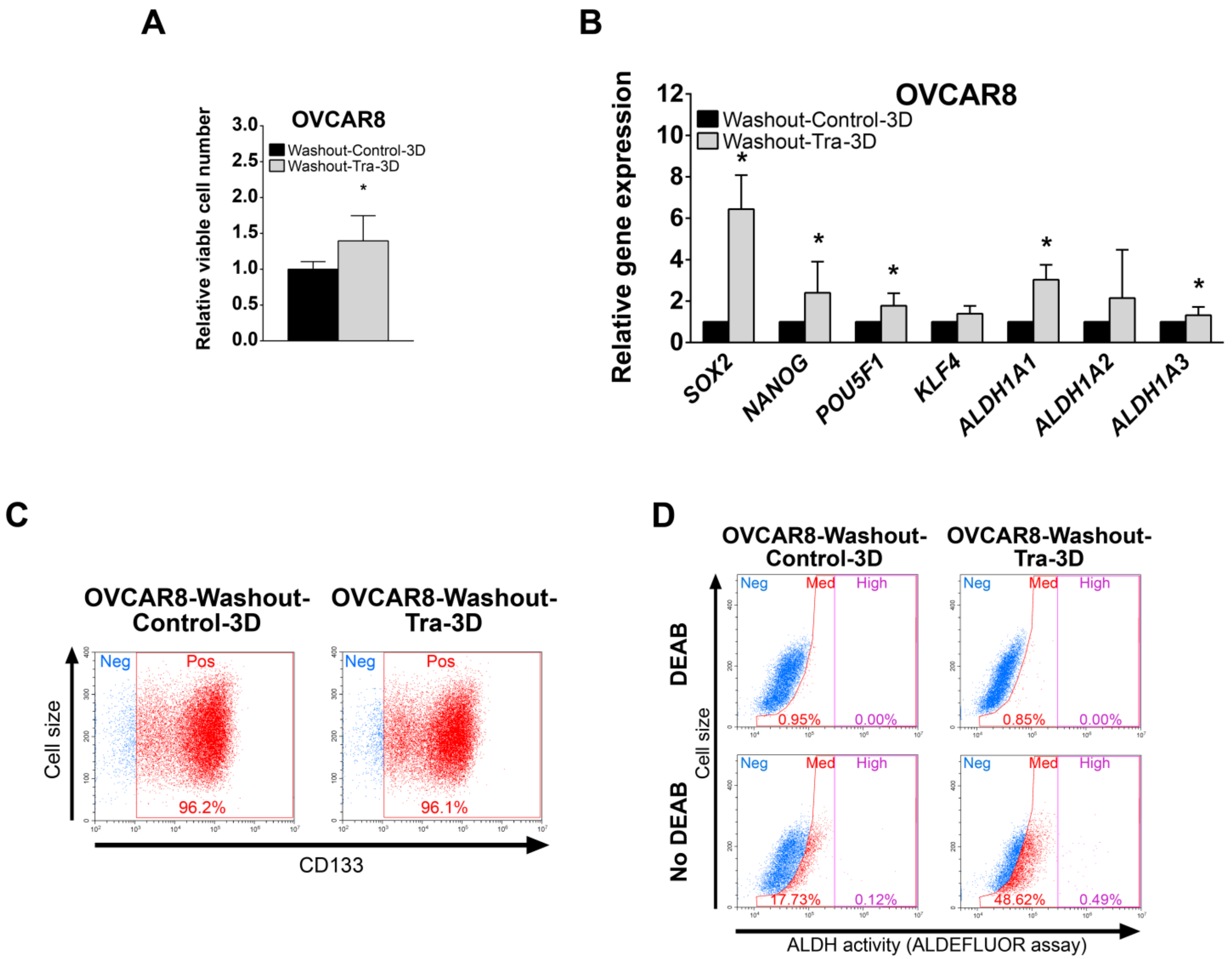

Figure S10. Stemness-related properties of OVCAR8-Washout cells grown in non-adherent 3D conditions. (A) Viable cell numbers after cultivation in non-adherent 3D conditions for 7 days. Data are normalized to "Washout-Control-3D" samples and presented as mean+S.D. (N=12, Mann-Whitney U-test). **(B)** Gene expression levels of stemness-related genes in cells grown in non-adherent conditions. Data are normalized to "Washout-Control" samples and presented as mean+S.D. (N=3, two-tailed Student's T-test with Welch's correction). **(C)** Expression of the CD133 surface marker in cells grown in non-adherent conditions. Gates indicate CD133-negative ("Neg") and CD133-positive ("Pos") cell subpopulations. **(D)** ALDEFLUOR analysis of cells after cultivation in non-adherent 3D conditions for 7 days. DEAB was used as ALDH inhibitor to define background ALDEFLUOR signal and set proper gates for ALDH-positive cells. Gates indicate cell subpopulations displaying negative ("Neg"), medium ("Med"), or high ("High") levels of ALDH activity. Tra – trametinib.

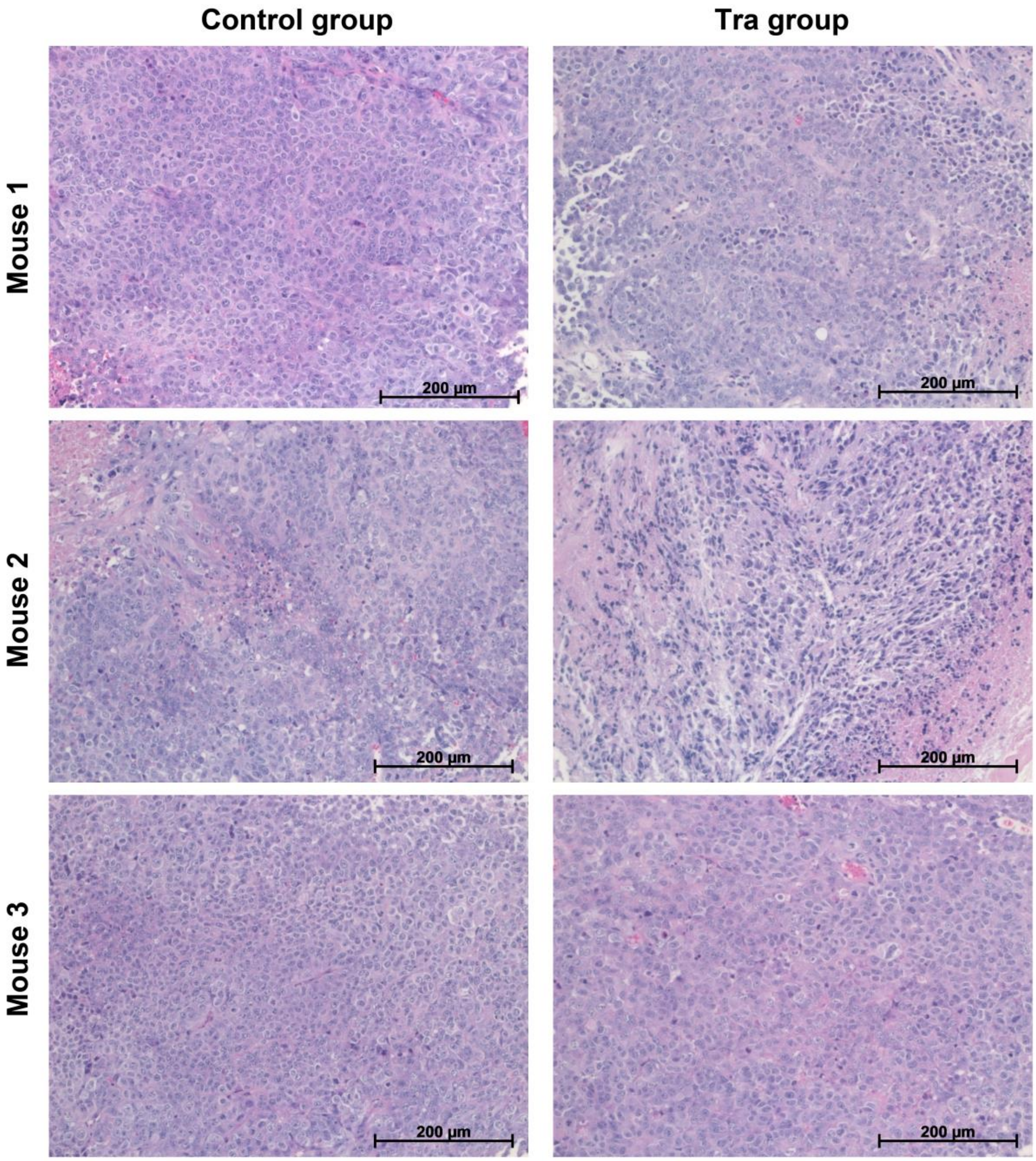

Figure S11. Hematoxylin and eosin staining of PEO4 xenograft tissue samples. Scale bars: 200 μm. Tra – trametinib.

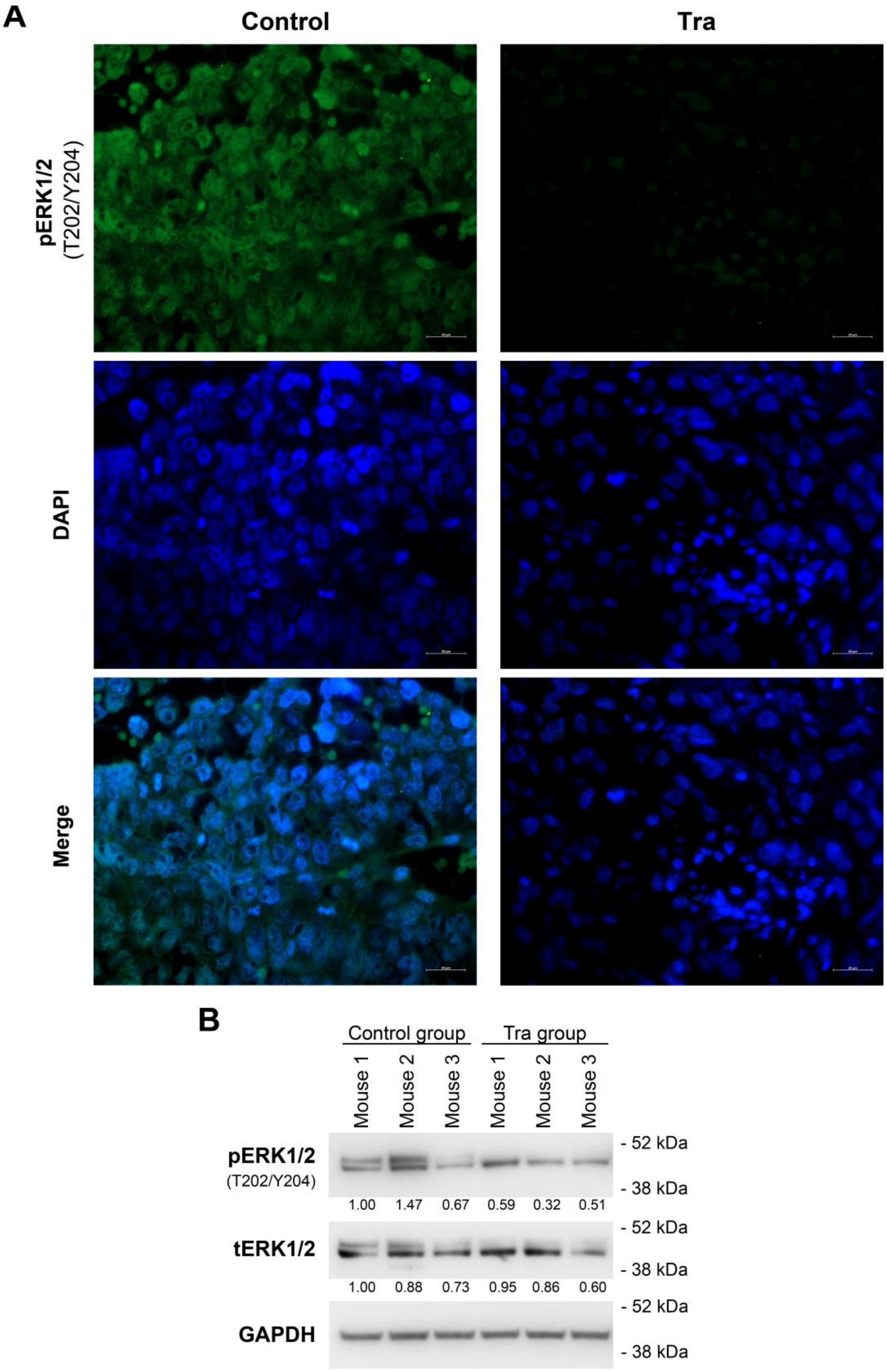

Figure S12. Effects of trametinib treatment on MEK1/2 pathway activity in HGSOC *in vivo*. (A) High-resolution images of immunofluorescent staining of phosphorylated ERK1/2 (green) in PEO4 xenograft tissue samples. Cell nuclei were counterstained with DAPI (blue). Scale bars: 20 µm. **(B)** Immunoblotting analysis of phosphorylated ERK1/2 levels in PEO4 xenograft tissue samples. Numbers under the bands represent relative intensity normalized to GAPDH levels and Control Mouse 1 sample. Tra – trametinib.

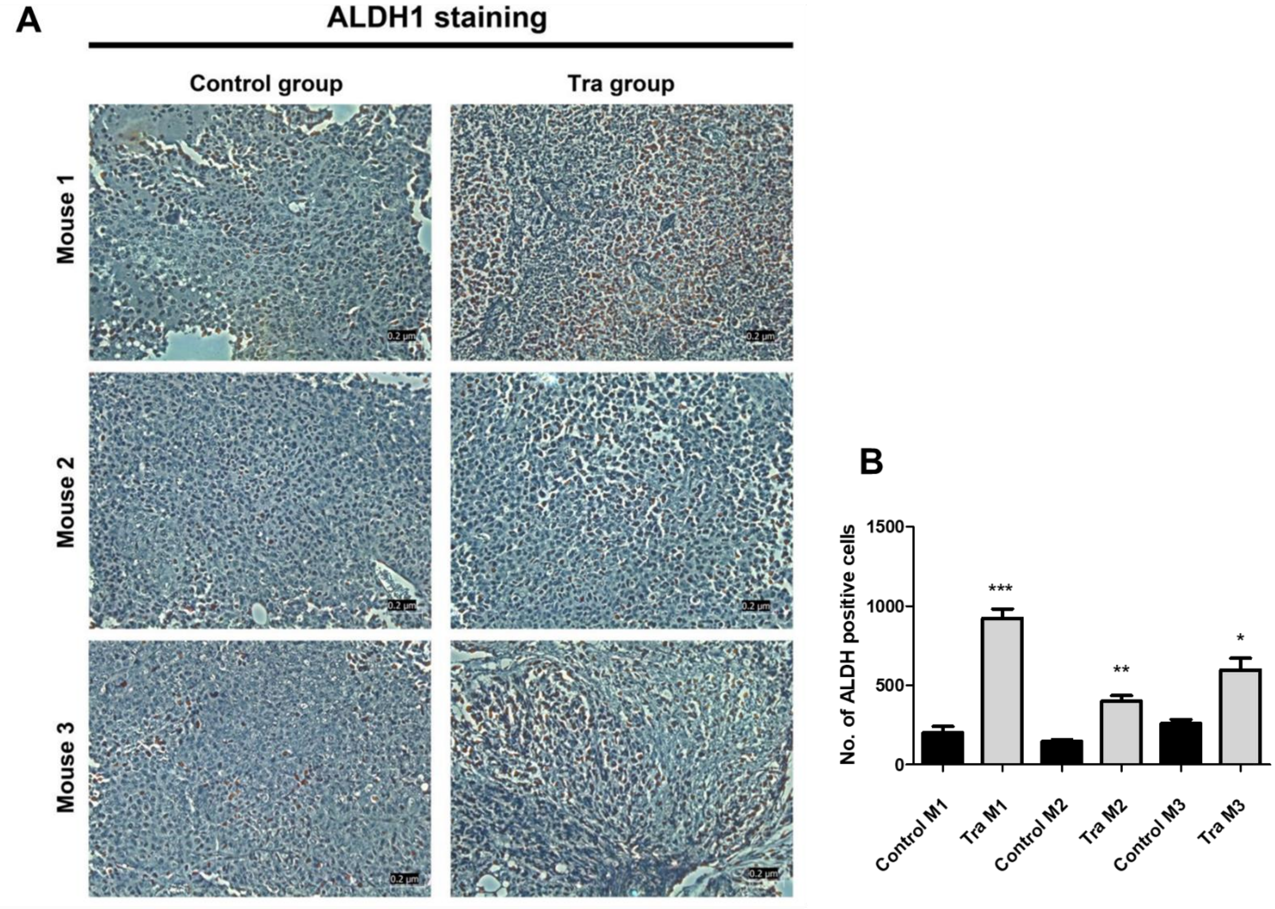

Figure S13. Effects of trametinib treatment on ALDH1 expression in HGSOC xenografts. A) Immunohistochemical staining of ALDH1 in PEO4 xenograft tissue samples with hematoxylin counterstaining. Scale bars: 0.2 µm. Tra –trametinib. B) No. of ALDH positive cells quantified by image J software. (N=5, two-tailed Student's T-test with Welch's correction). Tra – trametinib.

Uncropped Immunoblotting Images

Figure 2A.

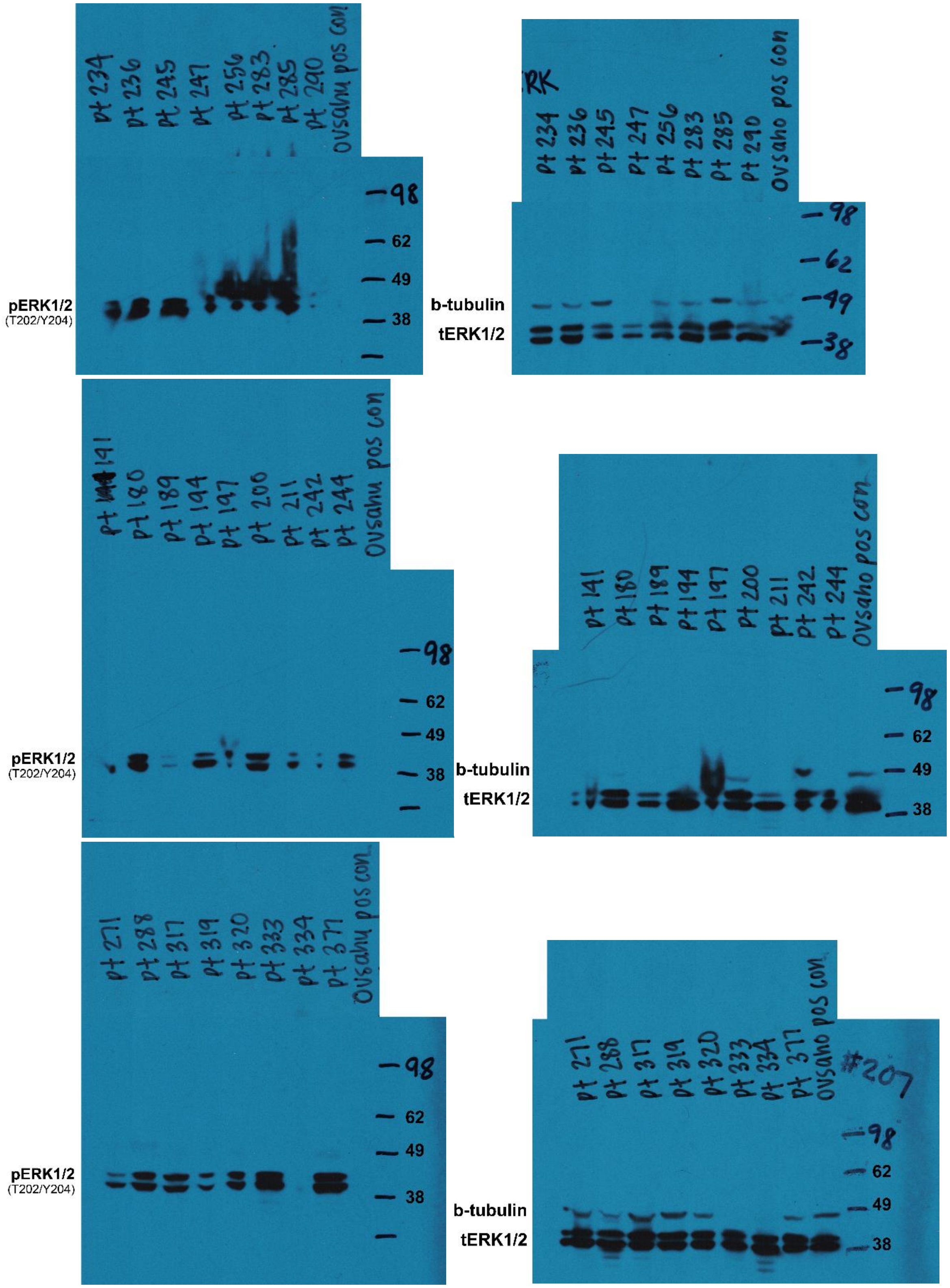

Figure 2A (Continued).

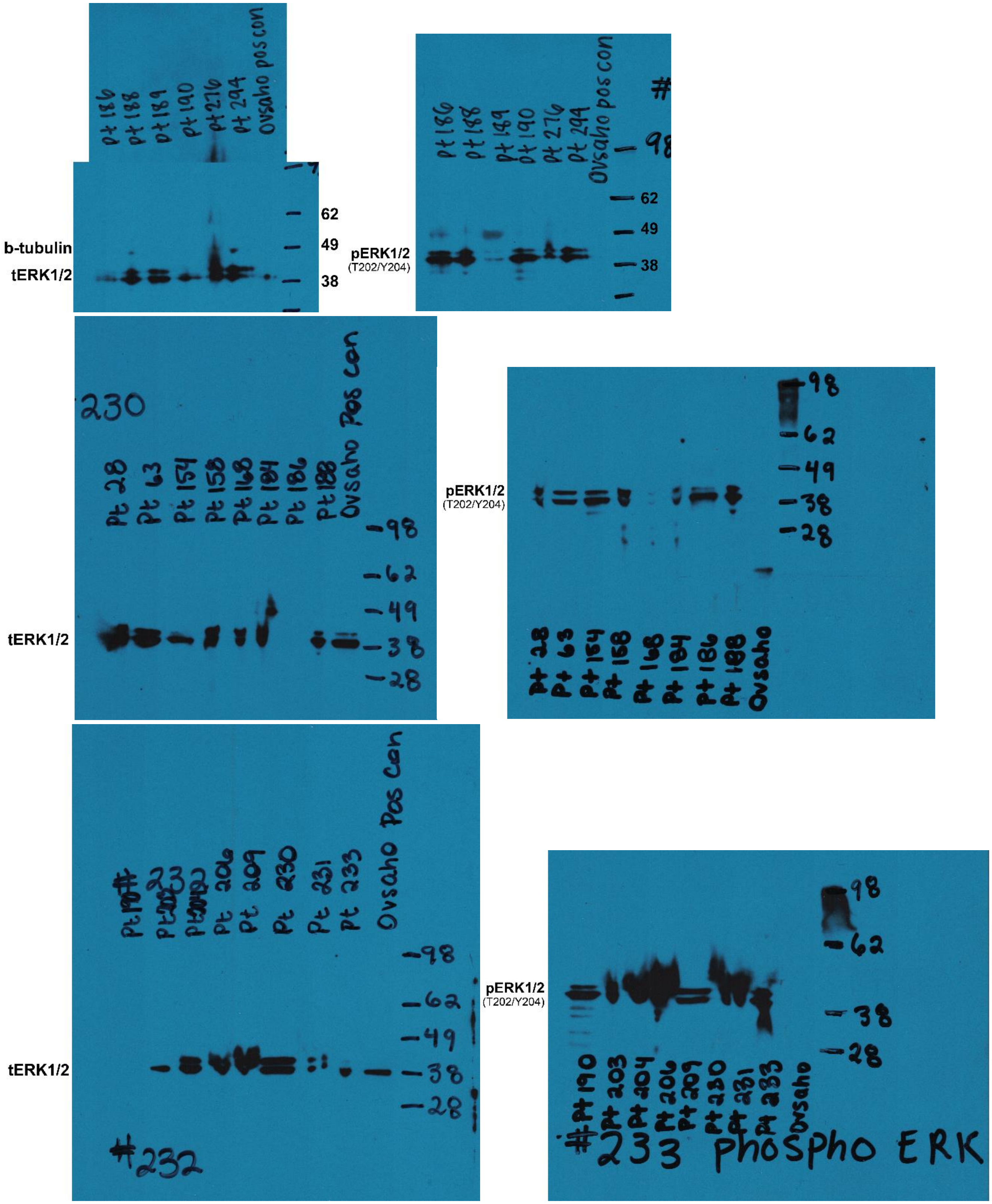

Figure 2A (Continued).

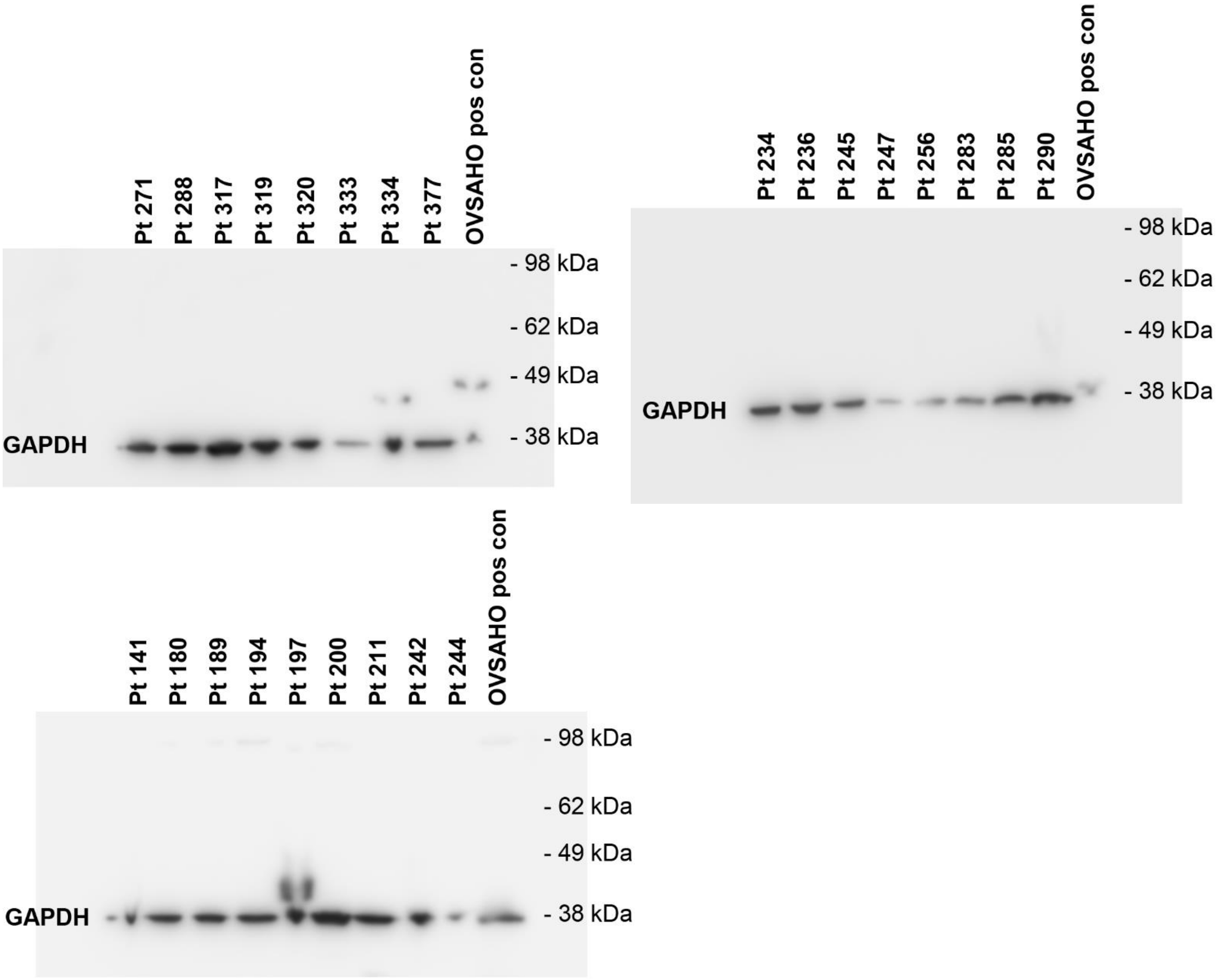

Figure 2C.

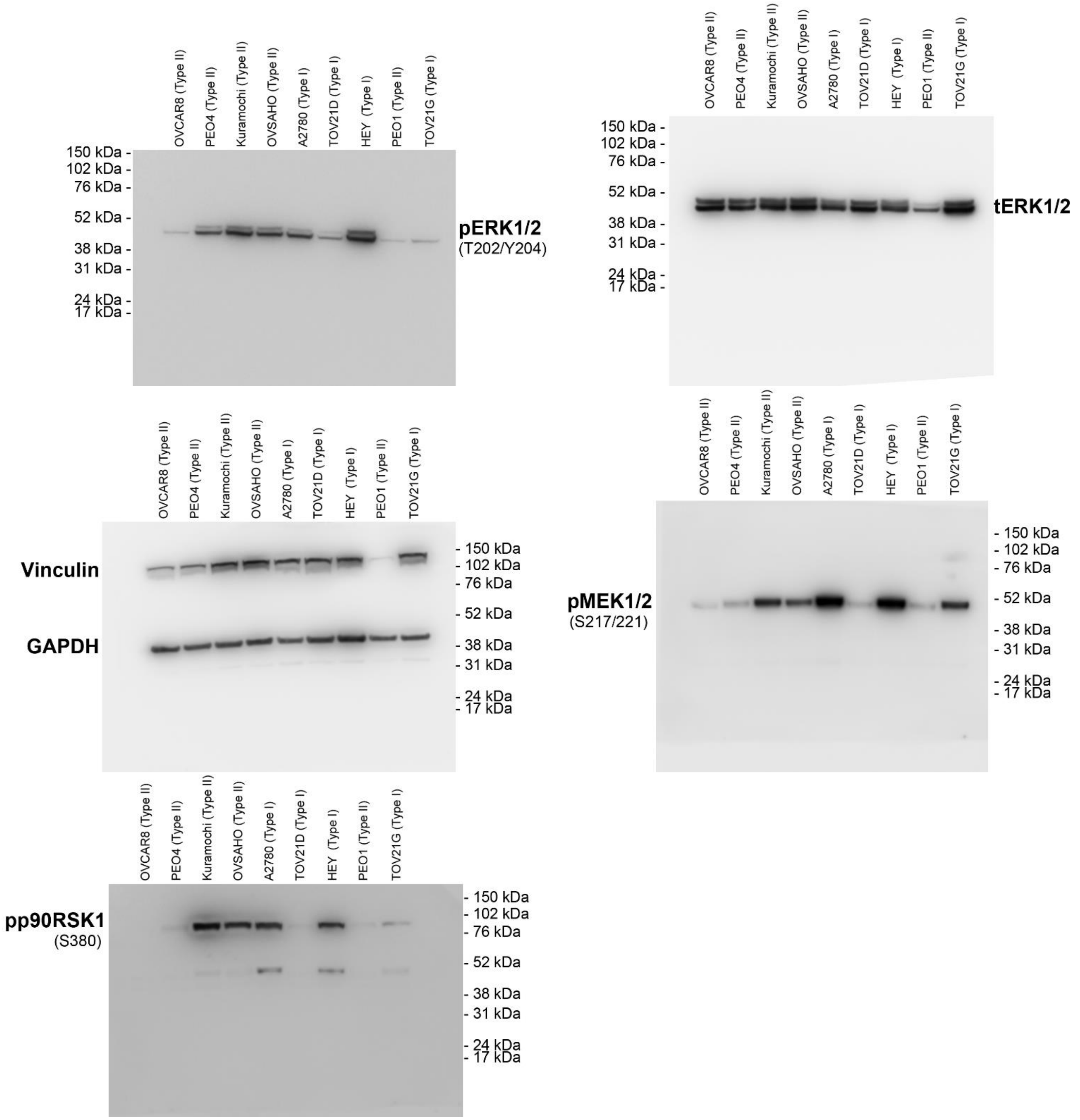

OVCAR8 (Type II)
PEO4 (Type II)
Kuramochi (Type II)
OVSAHO (Type II)
A2780 (Type I)
TOV21D (Type I)
HEY (Type I)
PEO1 (Type II)
TOV21G (Type I)
150 kDa
102 kDa
76 kDa
52 kDa
38 kDa
31 kDa
24 kDa
17 kDa
pERK1/2
(T202/Y204)
tERK1/2
Vinculin
GAPDH
pMEK1/2
(S217/221)
pp90RSK1
(S380)

Figure 3A.

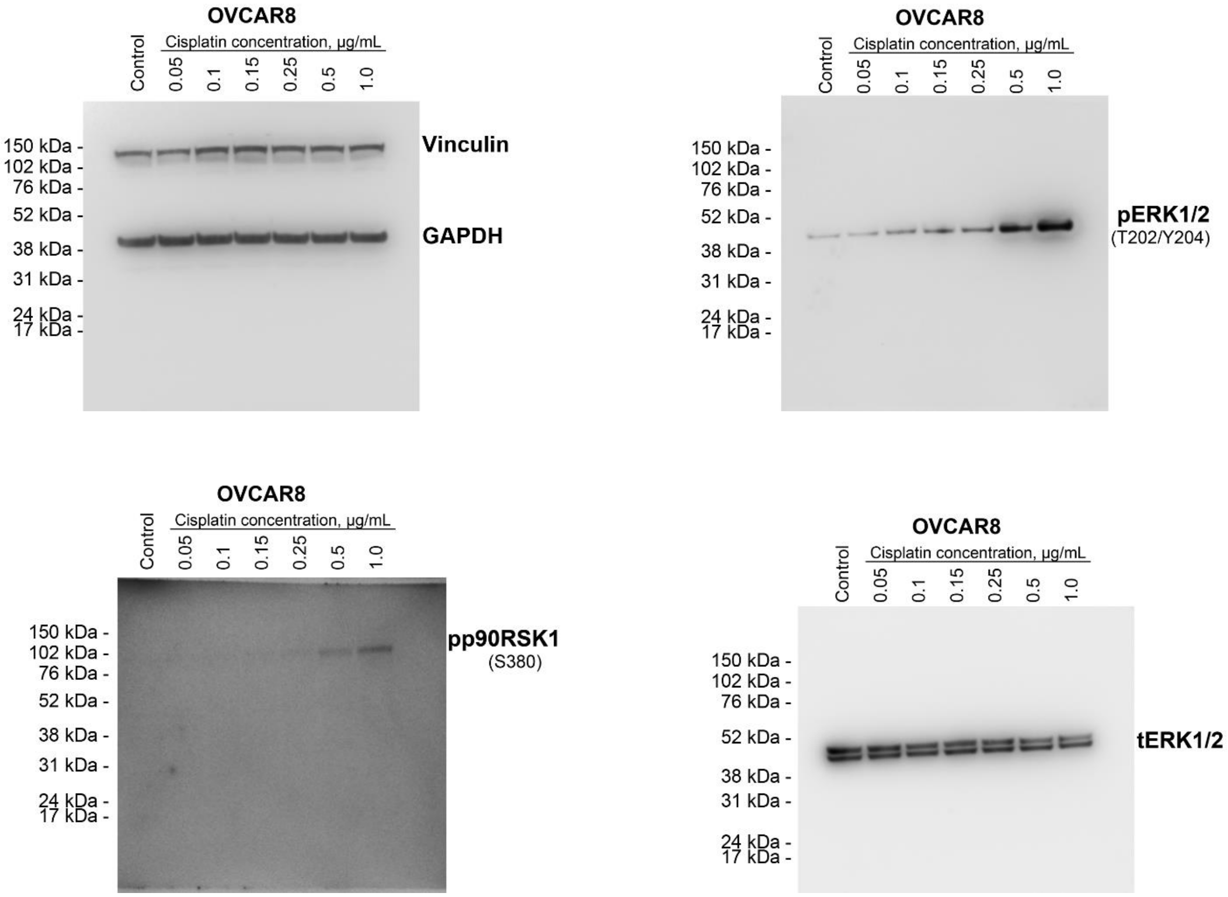
OVCAR8
Cisplatin concentration, µg/mL
Control
0.05
0.1
0.15
0.25
0.5
1.0
150 kDa -
102 kDa -
76 kDa -
52 kDa -
38 kDa -
31 kDa -
24 kDa -
17 kDa -
Vinculin
GAPDH
pERK1/2
(T202/Y204)
pp90RSK1
(S380)
tERK1/2

Figure 3A (Continued).

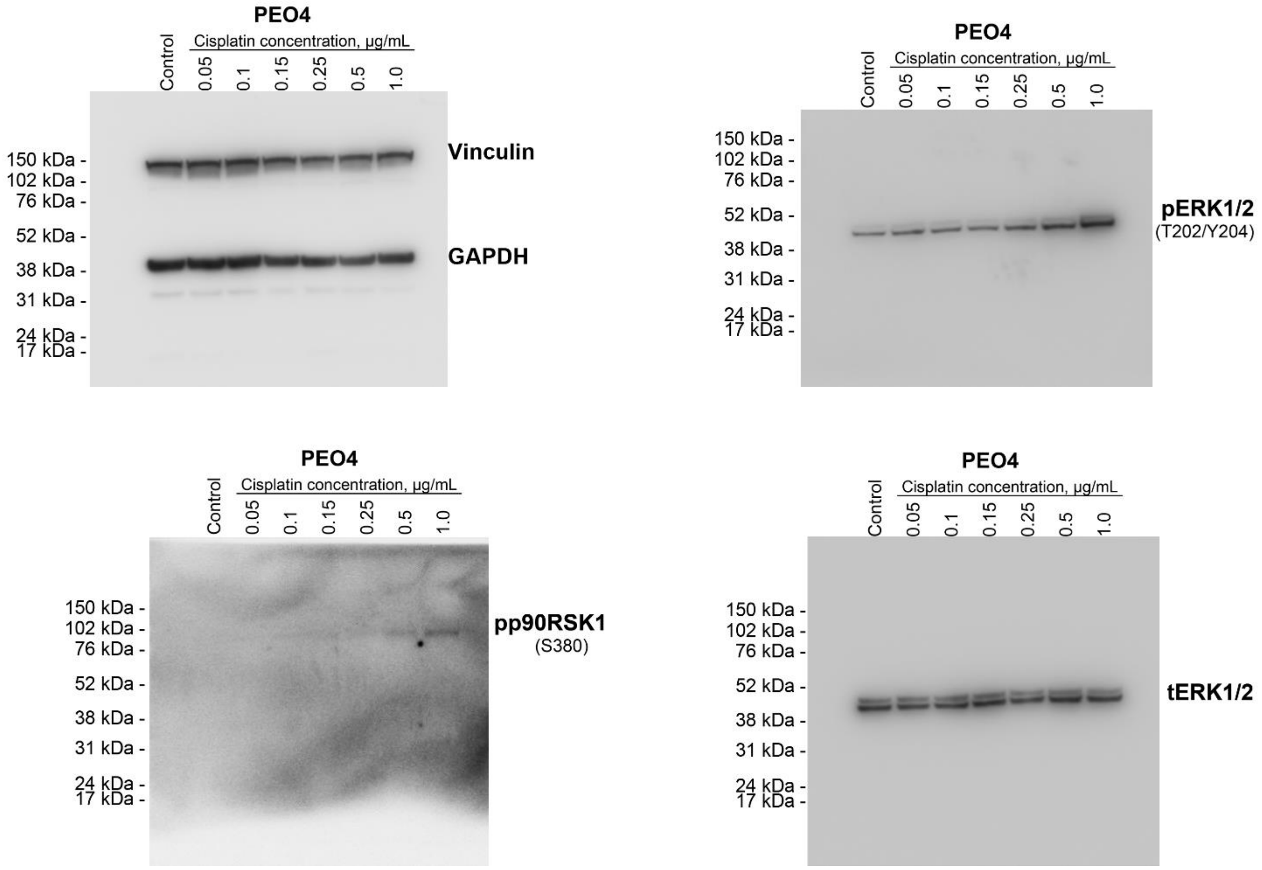

Figure 3B.

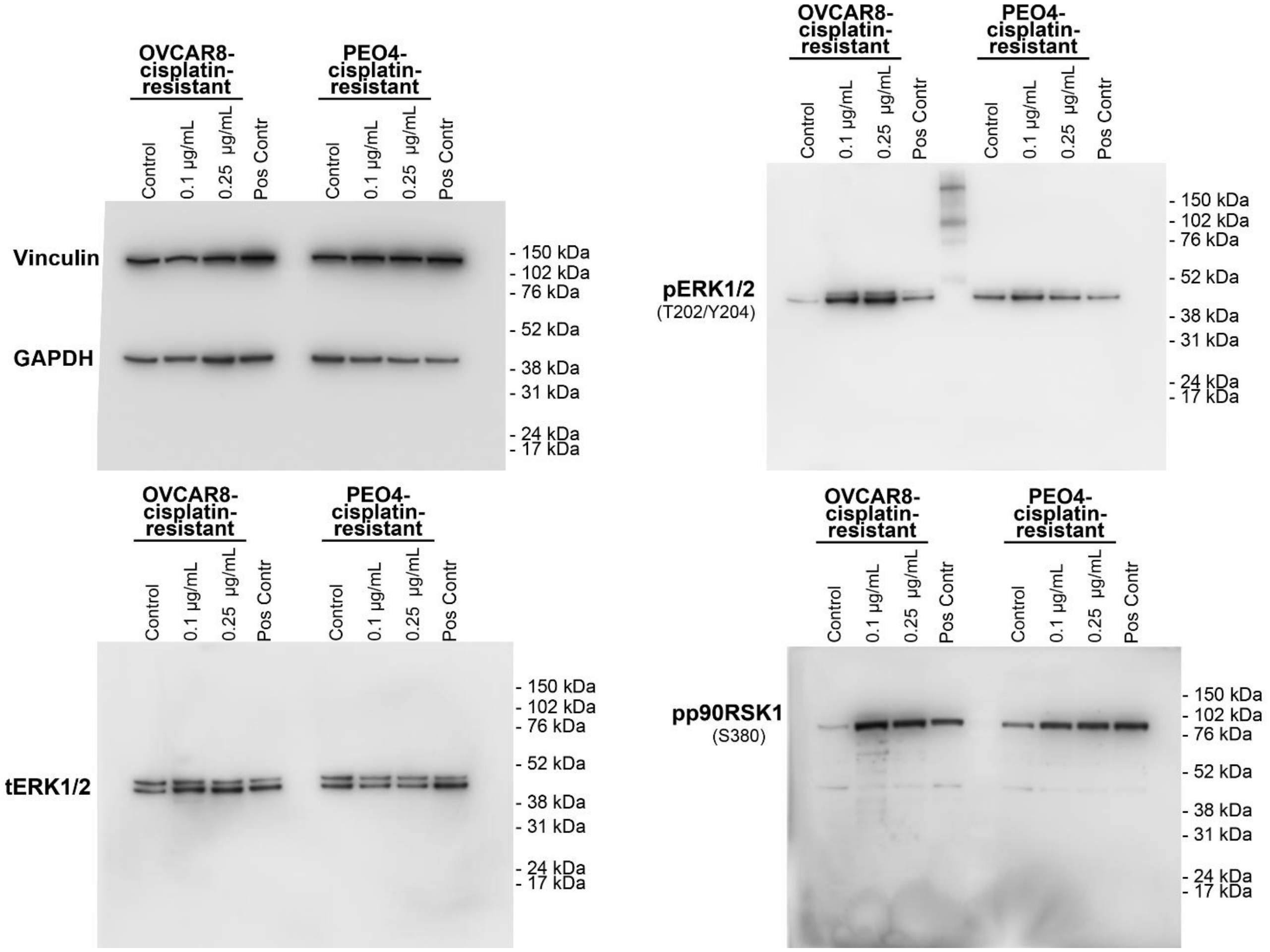
OVCAR8-cisplatin-resistant
PEO4-cisplatin-resistant
Control
0.1 μg/mL
0.25 μg/mL
Pos Contr
Vinculin
GAPDH
pERK1/2
(T202/Y204)
tERK1/2
pp90RSK1
(S380)
- 150 kDa
- 102 kDa
- 76 kDa
- 52 kDa
- 38 kDa
- 31 kDa
- 24 kDa
- 17 kDa

Figure 3C.

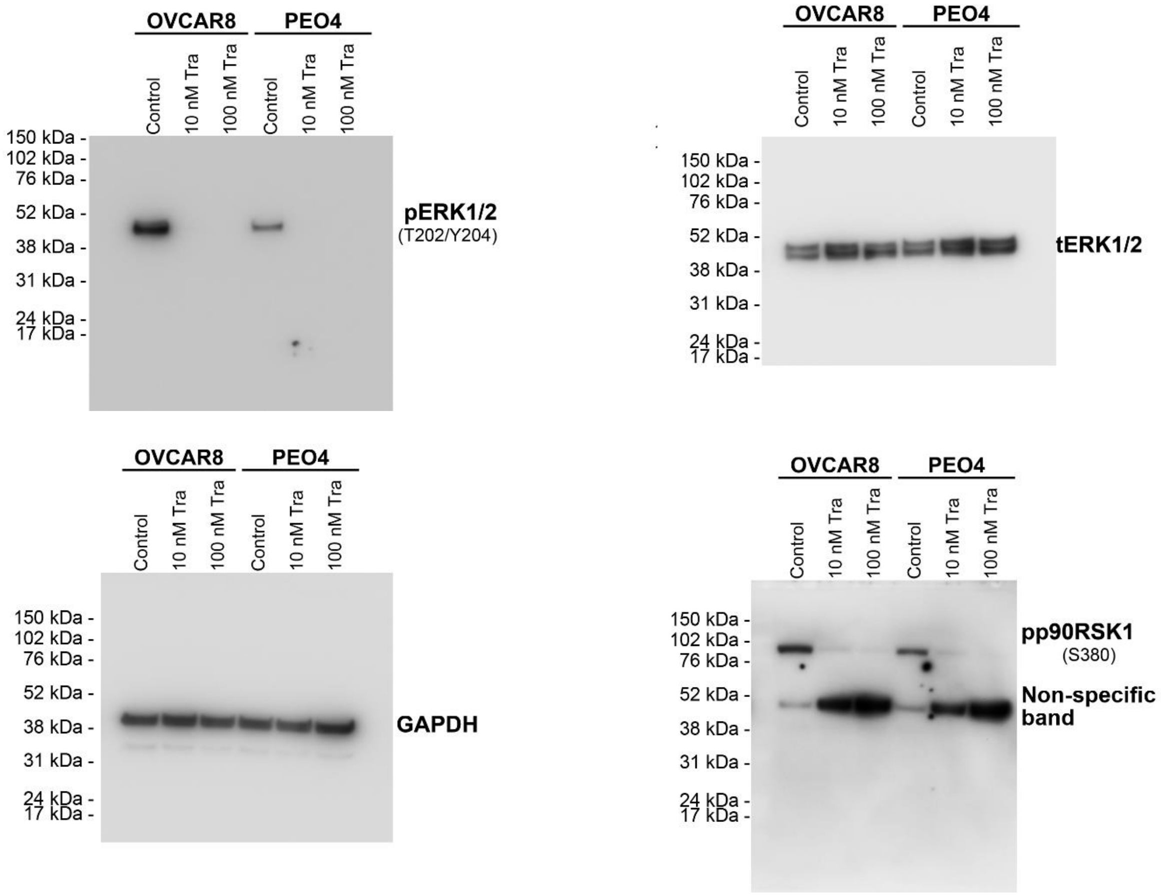
OVCAR8
PEO4
Control
10 nM Tra
100 nM Tra
150 kDa -
102 kDa -
76 kDa -
52 kDa -
38 kDa -
31 kDa -
24 kDa -
17 kDa -
pERK1/2
(T202/Y204)
tERK1/2
GAPDH
pp90RSK1
(S380)
Non-specific band

Figure 5C.

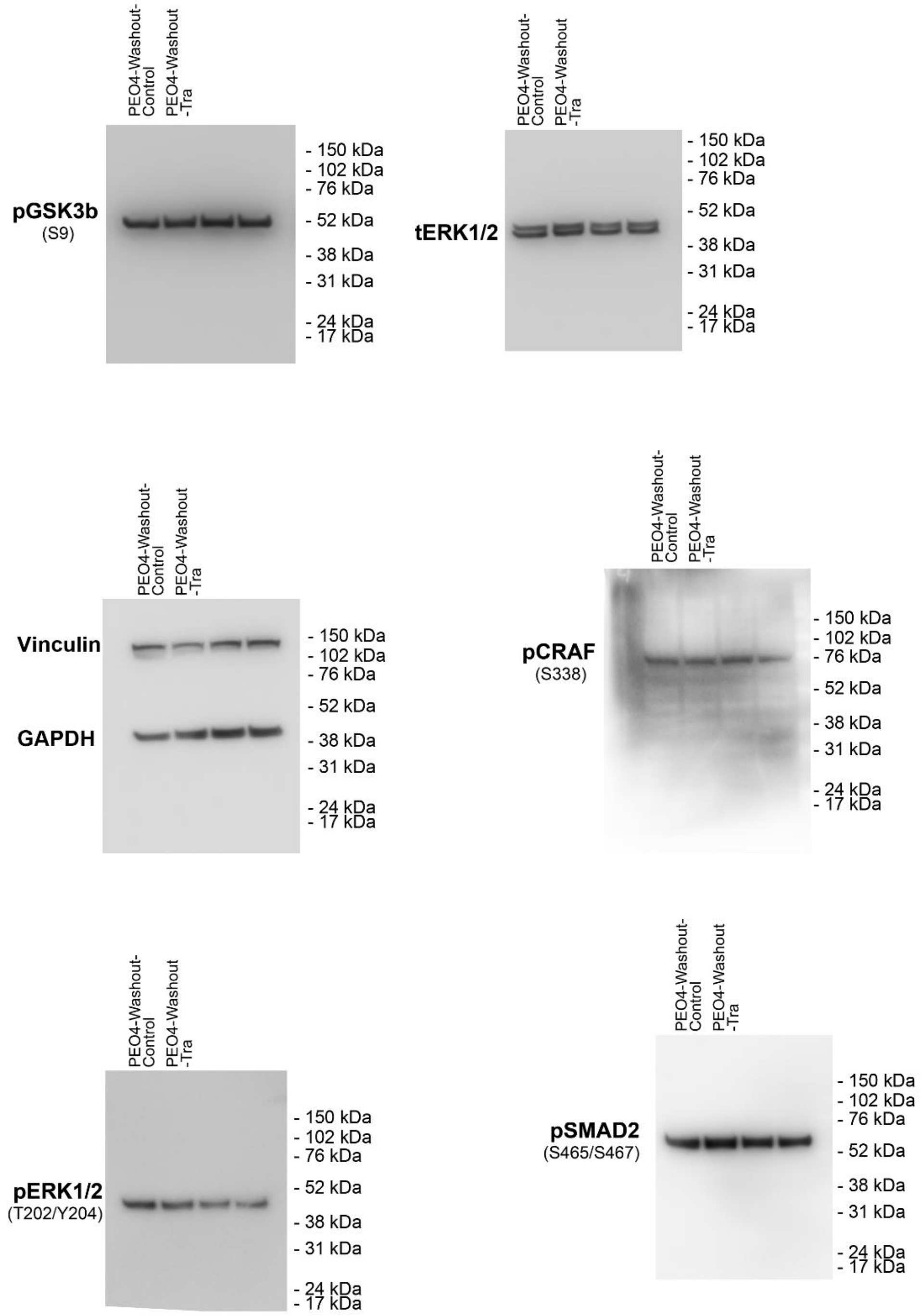
PEO4-Washout-Control
PEO4-Washout-Tra
pGSK3b
(S9)
tERK1/2
Vinculin
GAPDH
pCRAF
(S338)
pERK1/2
(T202/Y204)
pSMAD2
(S465/S467)
- 150 kDa
- 102 kDa
- 76 kDa
- 52 kDa
- 38 kDa
- 31 kDa
- 24 kDa
- 17 kDa

Figure S5.

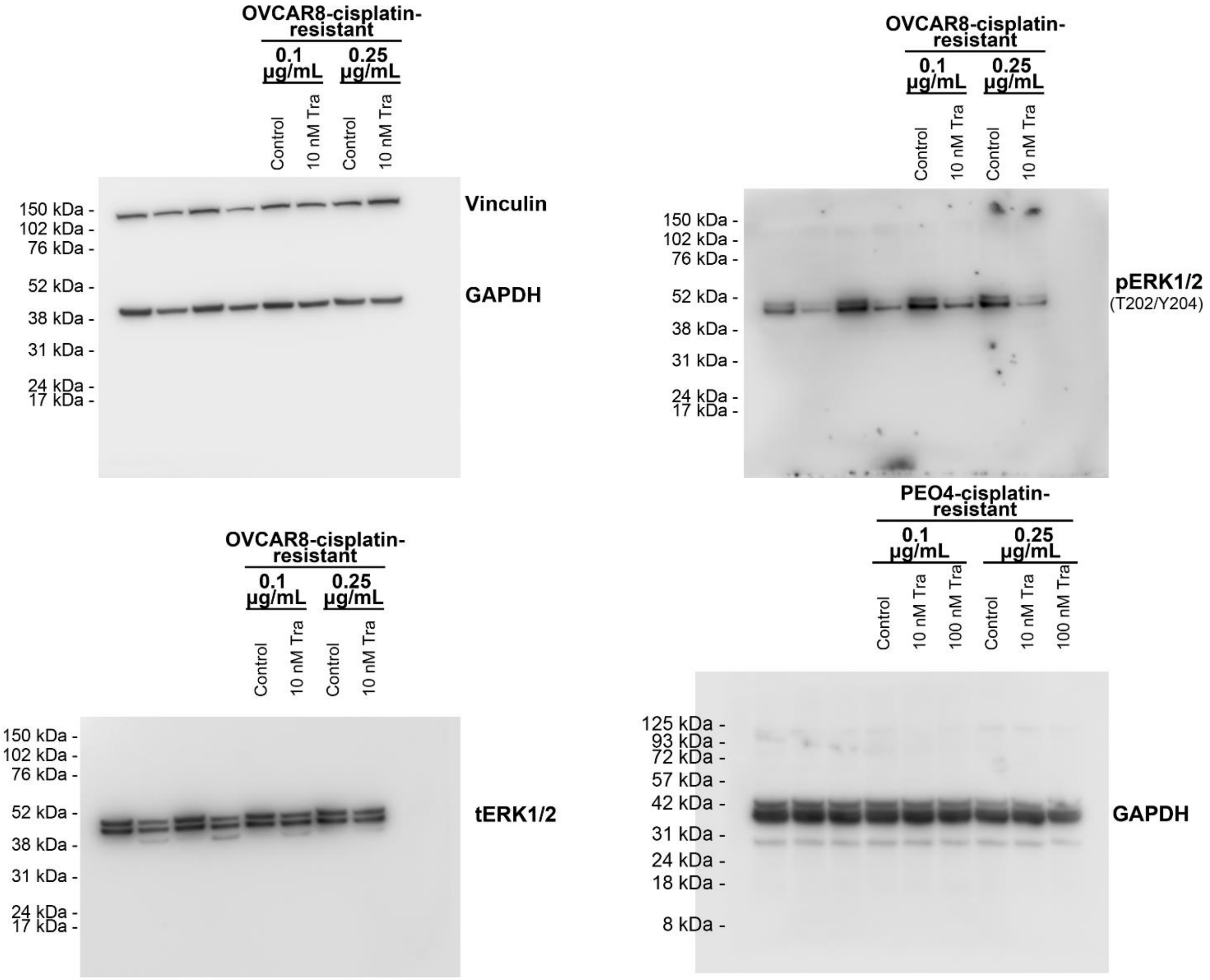

OVCAR8-cisplatin-resistant
0.1 µg/mL
0.25 µg/mL
Control
10 nM Tra
Control
10 nM Tra
150 kDa -
102 kDa -
76 kDa -
52 kDa -
38 kDa -
31 kDa -
24 kDa -
17 kDa -
Vinculin
GAPDH
OVCAR8-cisplatin-resistant
0.1 µg/mL
0.25 µg/mL
Control
10 nM Tra
Control
10 nM Tra
150 kDa -
102 kDa -
76 kDa -
52 kDa -
38 kDa -
31 kDa -
24 kDa -
17 kDa -
pERK1/2
(T202/Y204)
OVCAR8-cisplatin-resistant
0.1 µg/mL
0.25 µg/mL
Control
10 nM Tra
Control
10 nM Tra
150 kDa -
102 kDa -
76 kDa -
52 kDa -
38 kDa -
31 kDa -
24 kDa -
17 kDa -
tERK1/2
PEO4-cisplatin-resistant
0.1 µg/mL
0.25 µg/mL
Control
10 nM Tra
100 nM Tra
Control
10 nM Tra
100 nM Tra
125 kDa -
93 kDa -
72 kDa -
57 kDa -
42 kDa -
31 kDa -
24 kDa -
18 kDa -
8 kDa -
GAPDH

Figure S5 (Continued).

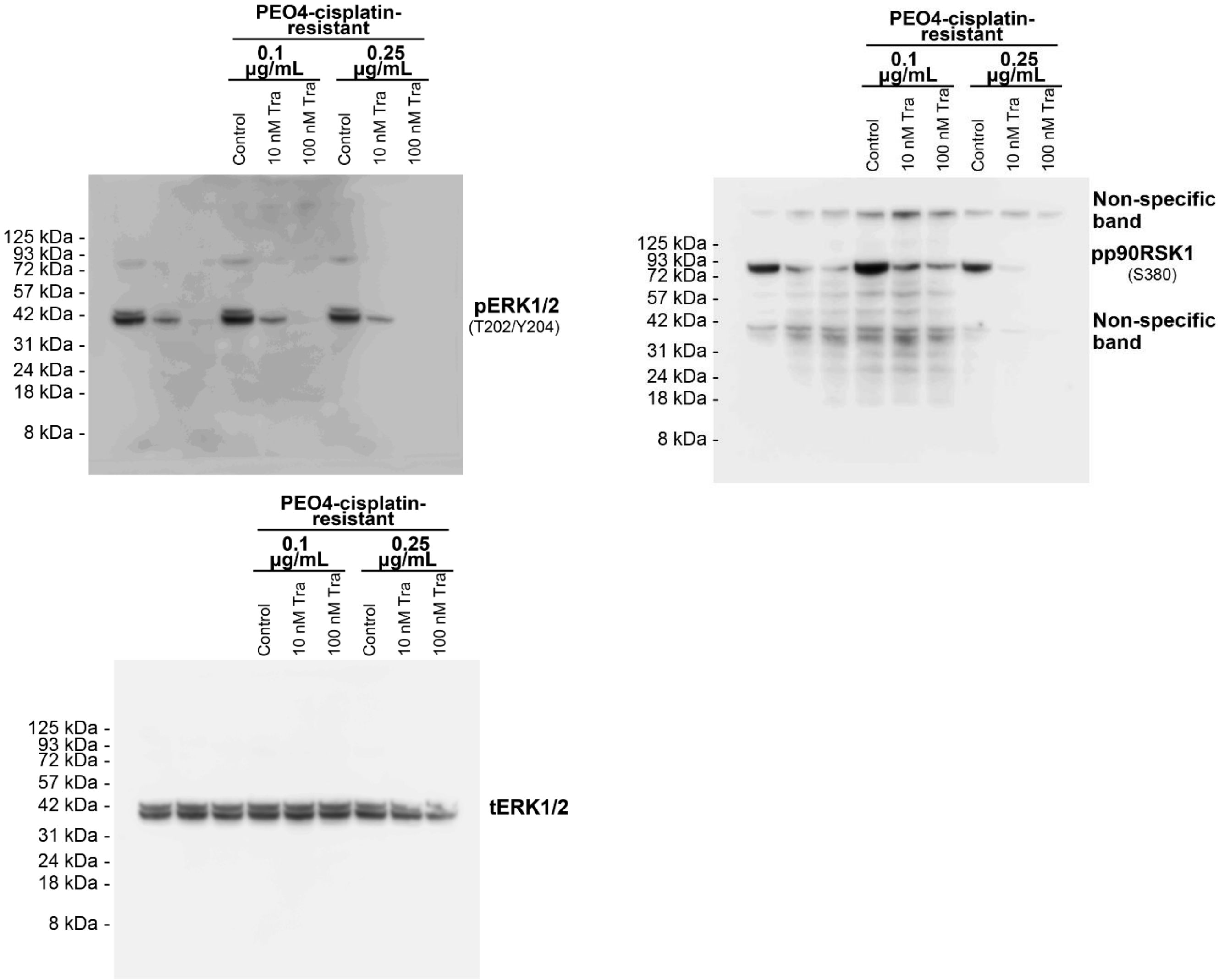

Figure S12B.

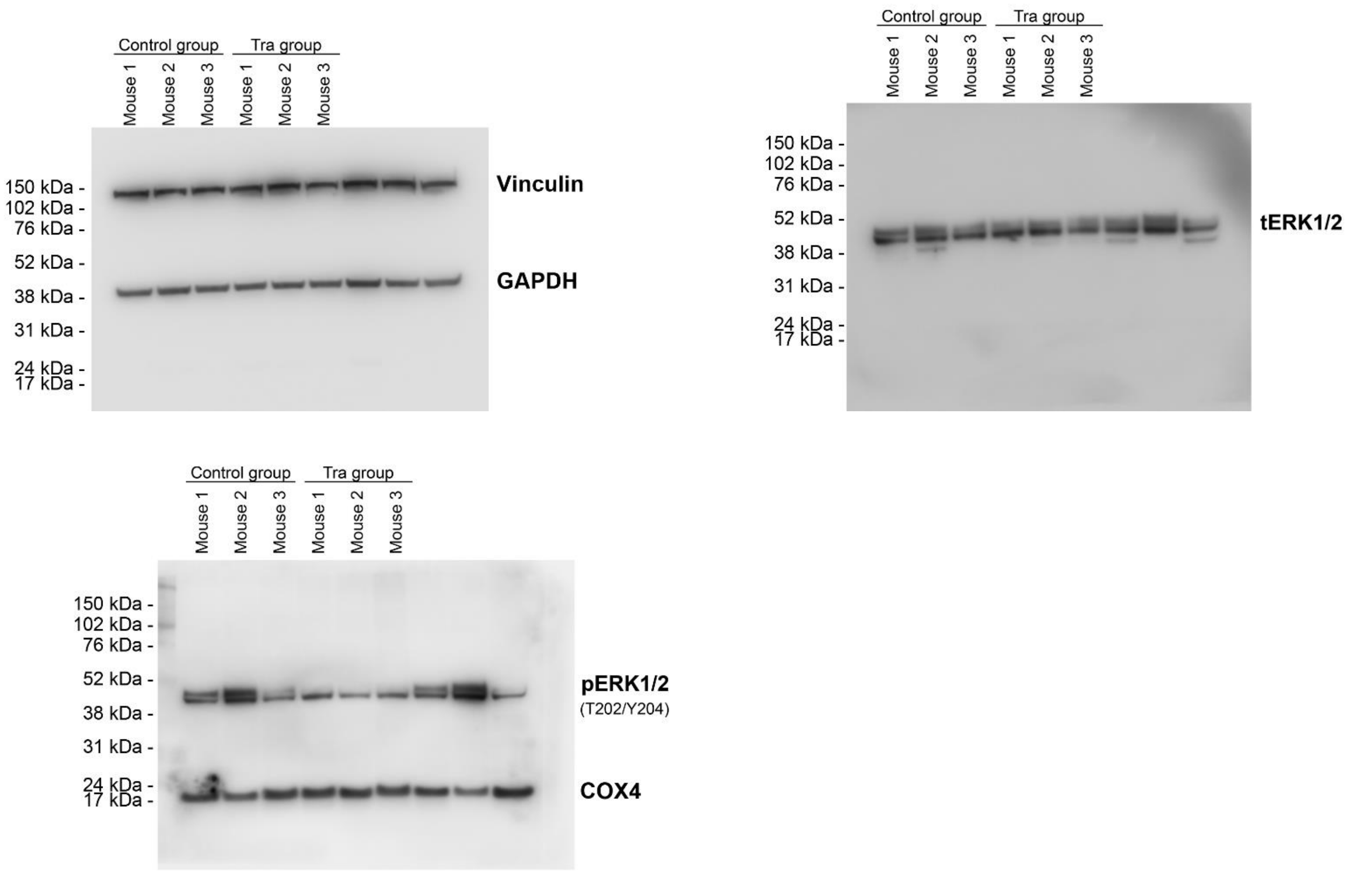
Control group
Tra group
Mouse 1
Mouse 2
Mouse 3
Mouse 1
Mouse 2
Mouse 3
150 kDa -
102 kDa -
76 kDa -
52 kDa -
38 kDa -
31 kDa -
24 kDa -
17 kDa -
Vinculin
GAPDH
tERK1/2
pERK1/2
(T202/Y204)
COX4
